# Optimization of durability characteristics of engineered cementitious composites combined with titanium dioxide as a nanomaterial applying RSM modelling

**DOI:** 10.1038/s41598-025-94382-7

**Published:** 2025-03-19

**Authors:** Naraindas Bheel, Imran Mir Chohan, Ahmed Saleh Alraeeini, Mamdooh Alwetaishi, Sahl Abdullah Waheeb, Loai Alkhattabi, Omrane Benjeddou

**Affiliations:** 1 Faculty of Engineering, Science and Technology, Indus University, Karachi, 75300 Pakistan; 2 Department of Mechanical Engineering, Universiti Teknologi PETRONAS, 32610 Bandar Seri Iskandar, Tronoh, Perak Malaysia; 3 Faculty of Engineering and Computer Science, Al-Nasser University, Sana’a, Yemen; 4 Department of Civil Engineering, College of Engineering, Taif University, P.O. Box 11099, 21944 Taif, Saudi Arabia; 5 Applied College, Umm Al-Qura University, P.O. Box 715, 21955 Macca, Saudi Arabia; 6 Department of Civil and Environmental Engineering, College of Engineering, University of Jeddah, 23890 Jeddah, Saudi Arabia; 7 Department of Civil Engineering, College of Engineering, Prince Sattam Bin Abdulaziz University, 16273 Alkharj, Saudi Arabia

**Keywords:** Cementitious composites, Titanium dioxide, Durability properties, Compressive strength, RSM modelling, And optimization, Materials science, Civil engineering, Nanoscale materials

## Abstract

Currently, chemical attacks, including acid attacks and sulphate attacks, pose a significant problem for the long-term durability of concrete infrastructures that encounter many types of water, including swamp water, marine water, sewage water, drinkable water, and groundwater. Therefore, the intention of this work is to enhance the durability and resistance of concrete against chemical attack by blending titanium dioxide (TiO_2_) as nanoparticles into designed cementitious composites. The purpose of current study is to obtain an appropriate TiO_2_ based on the cement’s weight and polyvinyl alcohol (PVA) fiber in composites using multi-objective optimisation. Thirteen mixtures comprising diverse combinations of variables (TiO_2_: 1–2%, PVA: 1–2%) were formulated utilising RSM modelling. Seven responses were assessed for these mixtures, including weight loss, compressive strength, expansion, a rapid chloride permeability test (RCPT) and a pH test. Analysis of variance, on the other hand, was utilised to construct and assess eight response models (one linear and six quadratics in nature). The R^2^ values for models spanning from 88 to 99%. The multi-objective optimisation generated optimal response values and ideal variable values (1% PVA and 1.5% TiO_2_). Experimental verification revealed that the predicted values correlated exceedingly well with the experimental data, with an error rate of less than 5%. The outcomes indicate that a 30% rise in compressive strength was noted when 1.5% TiO_2_ nanomaterial was incorporated into ECC. Furthermore, the expansion caused by sulphate attack decreases when TiO_2_ used as a nanomaterial increases in composites. Besides, when the concentration of TiO_2_ in ECC increased, the pH value, and weight loss caused by acid attack reduced. In addition, the RCPT is recorded reducing when the content of TiO_2_ increases but it increases with addition of PVA fiber. It has been shown that including 1.5% TiO_2_ and 1% PVA fiber yields the optimal results for the building sector.

## Introduction

Engineered cementitious composites (ECC) is one of the specific forms of high-performance fiber-reinforced cementitious composites (HPFRCC. They have a special mix of high ductility and an medium fiber volume percentage^1,2^. ECCs are designed to possess TiO_2_ toughness and hardening qualities, even in ordinary or harsh conditions. ECCs are composite constituents that are designed to help the concrete sector to optimise the usage of materials, reduce waste, offer cost effective and environmentally friendly advantages, and enhance the longevity of structures^3,4^. Furthermore, an Engineered Cementitious Composite is composite material for improving the durability of construction developments owing to its exceptional resistance to cracking resistance, impressive ductility, and ability to manage the width of crack^5^.This is due to the fact that ECCs have the ability to create stable and abundant microcracks, which greatly improve their durability in terms of ductility and tensile strength^6^. An ECC has a higher efficiency of approximately 3–5% compared to control mixture (CM) as a result of its high tensile strength^7–10^. The study findings indicate that the crushing strength of the material extends from 20 to 95 MPa, the tensile strength fluctuates between 4 and 12 MPa, while the compressive strain rises from 0.40 to 0.65%^11–14^.

Although ECC has certain advantages compared to conventional concrete, it also has several associated downsides. Initially, the drying shrinkage of the ECC mixture exceeded that of conventional concrete, leading to reduced eigenstrain in the concrete matrix under restraint. Because there are no large particles, ECC has a lower Young’s modulus than ordinary concrete. This leads to a larger strain when the material attains its maximum compressive strength (CS)^15^. Moreover, the presence of various naturally occurring and man-made substances often exposes concrete projects to corrosive environmental conditions. Sulphate is a significant contributor to the deterioration of mortar and concrete construction^16^. When sulphate particles mix with the calcium hydroxide and calcium aluminate hydrate that are already in Portland cement (PC), certain things happen^17,18^. Ettringite and gypsum are responsible for the expansion and fracturing of concrete, leading to a brittle and less durable surface^19,20^.

Sulphate breaks down the important parts of wet Portland cement, specifically calcium silicate and calcium hydroxide, which weakens the bond between concrete particles. This results in a reduction in the strength and durability of the concrete^21^. Acid exposure in concrete is widely regarded as a secondary threat. Acid can corrode concrete in many ways. Acids present in soil or underwater, whether they occur by nature or are a result of waste from industrial process disposal, could harm the concrete structures located beneath the surface. Industrial applications may potentially experience acid leakage and inadvertent spills^22^. The technique for carrying out an acid attack varies due to the type of acid used. Nitric acid, sulfuric, hydrochloric, and carbonic are the main encountered acids related to concrete. The ultimate result is the disintegration of calcium hydroxide and cement hydrates into calcium salts, leading to the deterioration of exposed concrete. Furthermore, the acid attack differs the sulphate attack in that it does not result in considerable expansion during breakdown. In contrast to the hydration products of cement, the carbonaceous substantial is prone to acid assault^23^. Unlike other acids, sulfuric acid (H_2_SO_4_) can dissolve and expand concrete. According to multiple scientists, concrete treated with sulfuric acid decreases weight. When sulfuric acid chemically combines with calcium silicate hydrate gel (CSHG) and calcium hydroxide, it produces gypsum. The process by which concrete dissolves and expands is referred to as gypsum corrosion. Gypsum corrosion causes a decrease in concrete’s alkalinity, which in turn leads to microbiological corrosion. Corrosion produces an inferior compound lacking cementitious properties, leading to rapid deterioration, weakening, and, in severe cases, complete disintegration and collapse of the concrete structure. Gypsum can undergo a subsequent series of reactions described in the section on sulphate attack during the second stage of sulfuric acid-induced concrete corrosion, leading to the formation of ettringite or/ and Thomasite. Though, ettringite and Thomasite are not dependable in an acidic atmosphere^24,25^, hence the main outcome would be gypsum when the sulfuric acid attack.

Researchers investigated two methods to improve concrete’s acid resistance: reducing its permeability and including alternative mineral fillers to decrease calcium hydroxide production when there is a hydration of Portland cement^26^. Scientists have developed unique mortars and concrete by replacing certain weight proportions of cement with additives, showing encouraging outcomes in resisting sulphate attacks^27,28^. Concrete uses mineral admixtures, which include nanoparticles, to mitigate the harmful effects of sulphate and acid assaults^29,30^. The efficiency of a pozzolanic reaction is evident due to its inverse correlation with the surface area of amorphous nanoscale particles. Generally, the incorporation of pozzolan enhances the density and resistance of concrete to chemical attacks. However, it also reduces or eliminates the free and leachable calcium hydroxide^31^. Emerging nanotechnology advancements hold great potential for advancing nanoscale materials. Nanotechnology has facilitated the manipulation of matter at the atomic scale, thereby empowering researchers to exert control over the qualities of the resulting product. Studies have demonstrated that nano-engineered materials surpass their larger-scale equivalents in performance because of their small size^32–35^. Titanium dioxide (TiO_2_) has emerged as a revolutionary nanomaterial in the construction sectors, offering a solution to the need for high-performance materials at a reasonable cost. Cementitious composites widely use Titanium dioxide (Titania) nanoparticles owing to their stable physical and chemical attack, strong photochemical activity, and cost-effectiveness^36–39^. These nanoparticles are particularly valuable because they accelerate the hydration development by occupying microscale gaps and offering sites for the development of hydration products^40–42^. Meng et al.^43^ found that incorporating titanium dioxide (TiO_2_) nanoparticles at a concentration of 0.05% into cement significantly enhanced its compressive strength by 45% during the early stages of curing. This happened because the growth of C–S–H sped up and the number of pores went down after nanoparticles were added as nanofillers. Jayapalan et al.^44^ discovered that TiO_2_ nanoparticles induced the formation of nucleation sites within PC hydration products. These particles accelerated the reaction by reducing the energy barrier, which is the lowest quantity of energy needed for the reaction to occur. Zhang et al.^45^ verified that including TiO_2_ nanomaterial in cement mortar has a substantial impact on its compressive strength. The process expedites cement hydration and enhances the pore-refining action to achieve this. Feng et al.^46^ found that substitution of titanium nanoparticles resulted in an enhancement of the 28-day flexural strength of cement paste. The addition of nanomodification enhanced the paste microstructure by augmenting the cementitious phase, reducing porosity and internal micro-cracks and defects, attaining a denser microstructure with reduced roughness, and creating needle-shaped nanoparticles. The results show that using TiO_2_ as a nanomaterial can effectively lessen the problems that come with putting an ECC into wide use. It can also effectively counteract the negative effects of acid attack and sulphate attack that were seen in this study. The principal goal of this investigation was to find out how strong an ECC was when it had different amounts of PVA fibers and TiO_2_ as a nanomaterial, how well it resisted acid and sulphate attacks, and how quickly chloride could pass through it. They accomplished this by employing RSM modelling and optimisation techniques.

## Experimental program

### Materials

Portland cement (PC), which complied with the ASTM C150^47^ standard, served as the binding agent for ECC production. The investigation classified the collected fly ash (FA) as class F fly debris, which conforms to ASTM C618 specifications^48^. Table 1 presents the chemical composition for FA and PC. Furthermore, the experiment incorporated TiO_2_ powder as a nanomaterial into malleable concrete. The particle dimensions vary from 10 to 50 nm. The experiment employed a third-generation superplasticizer to uniformly distribute the nanoparticles, preventing any adhesion or aggregation upon combination with water^49^. However, river sand was used as fine aggregates, and it was sieved (lesser than about 4.75 mm) prior used in mixture. The polyvinyl alcohol (PVA) fiber was used by ECC’s volume fraction to determine the mixture’s fiber content. To alter the properties of the interface between the fiber and composites, a 1.2% bulk coating of oil was useful to the fiber surface. Additionally, a superplasticizer (SP) was added into the ECC. The SP possessed a specific gravity about of 1.08 and a pH of approximately 6.2. One percent by weight of the PC was introduced to the mixture of ECC. Furthermore, the TiO_2_ investigation used potable water for the blending and curing functions.

**Table 1:** XRF of PC and FA.

Materials	Compound (%)									Specific gravity	Blaine fineness (m^2^/kg**)**
	SiO_2_	Al_2_O_3_	Fe_2_O_3_	MnO	CaO	TiO_2_	Na_2_O	K_2_O	T_2_O		
PC	20.76	5.54	3.35	–	61.4	2.48	0.19	0.78	–	3.15	290
FA	57.01	20.96	4.15	0.033	9.79	1.75	2.23	1.53	0.68	2.38	325

### Mix proportions of TiO_2_-ECC

It was adopted to conduct various trial mixes to obtain the targeted compressive strength more than 45 MPa and then blended with several concentrations of TiO_2_ and fiber. Moreover, the RSM modelling used to generate the runs by choosing TiO_2_ and PVA fiber as input parameters to fulfil the ECC requirements of this investigation. The two input factors under investigation were TiO_2_ and PVA at three different concentrations: 1%, 1.5%, and 2% by volume fraction of PVA fiber and 1%, 1.5, and 2% by weight of PC, respectively. In contrast, the water-to-binder ratio for all ECC was 0.30, with variations in percentages observed for ECC-M45, which was the most expansively used ECC in many studies. In this experimental study^50,51^, and a SP comprising 1.0% by mass of PC were utilised. Furthermore, the thirteen experimental trials were generated through the implementation of the central composite design (CCD) approach in RSM. The mixtures consisted of diverse concentrations and combinations of the input variables, in addition to five arbitrary iterations of each parameter, as shown in Table 2. The identical combinations were utilised to ascertain the experiment’s validity and mitigate the risk of potential variations. Additionally, the RSM assesses the effect of the interrelation among input elements on the resulting responses. As responses, compressive strength, tensile strength, apparent porosity, acid attack, sulphate attack, and rapid chloride penetration test (RCPT) were analysed.

**Table 2:** Mix Proportion of TiO_2_-ECC.

ID (MIX)	Materials in percentage (%)		Quantity of constituents utilized in ECC (kg/m^3^)					
	TiO_2_	PVA fiber	PC	TiO_2_	Fly ash	Sand	SP	Water
CM1	0.00	1	545	0.0	650	450	5.45	164
CM2	0.00	1.5	545	0.0	650	450	5.45	164
CM3	0.00	2	545	0.0	650	450	5.45	164
M1	1	1	545	5.45	650	450	5.45	164
M2	1.5	1.5	545	8.17	650	450	5.45	164
M3	1	1.5	545	5.45	650	450	5.45	164
M4	2	1	545	10.90	650	450	5.45	164
M5	1.5	1	545	8.17	650	450	5.45	164
M6	1.5	1.5	545	8.17	650	450	5.45	164
M7	1.5	1.5	545	8.17	650	450	5.45	164
M8	1	1	545	5.45	650	450	5.45	164
M9	2	1.5	545	10.90	650	450	5.45	164
M10	1	2	545	5.45	650	450	5.45	164
M11	1.5	2	545	8.17	650	450	5.45	164
M12	2	1.5	545	10.90	650	450	5.45	164
M13	2	2	545	10.90	650	450	5.45	164

### Sample preparation and testing methods

This experimental study uses the mixing configuration of TiO_2_-ECC composites. The process of simultaneously incorporating the FA, PC, and sand into the dry concrete mixer lasted nearly two minutes. After that, a high-speed agitator vigorously combined TiO_2_ and superplasticizer with water for a duration of three to five minutes. As previously stated, the experiment utilised the super-plasticizer in order to prevent potential agglomeration, enhance workability, and facilitate uniform dispersion by capitalising on the cohesive characteristics of the nanoparticles^52,53^. Once the ECC mixture and nanoparticles were prepared, the nanoparticle mixture was systematically poured into the rotary mixer. In this manner, the complete nanoparticle mixtures were consumed until they were thoroughly incorporated into the concrete mixture. Following this, the PVA fiber was incorporated into the revolving mixer with caution for an additional duration of 2–3 min, or until it was ensured that the fibers were evenly distributed across the mixture. In order to restore the flowability of mixtures containing a high concentration of TiO_2_ and render them functional, SP was introduced. A diverse range of specimens were fabricated from the fresh concrete mixture in order to conduct tests on its sulphate attack, acid attack, compressive strength, and RCPT.

The cube specimens having (50 mm × 50 mm × 50 mm) dimension were fabricated using TiO_2_-ECC in agreement with the BS EN 12390-3^54^ guidelines to study the material’s compressive strength after 28 days. The direct tensile test used dog-bone-shaped samples measuring 420 mm × 120 mm × 30 mm, executed in accordance with the JSCE^55^ protocol. The assessment was performed at a regulated loading frequency of 0.15 mm/minute using a 200 kN-capacity Universal Testing Machine (UTM) fitted with integrated linear variable differential transformers (LVDTs). During the evaluation, a computer gathered and analysed real-time test data. Furthermore, the compressive strength and weight loss of the cubical specimens (50 mm × 50 mm × 50 mm) caused by acid attack were assessed. To assess the weight loss, all specimens were submerged in a 10% sulfuric acid (H_2_SO_4_) mixture, while their monthly reduction and loss of compressive strength were monitored. After 28 days of standard curing, the outer surface humidity from all concrete sample were also removed and cleaned away. Through pH analysis, the pH value of 3 samples of ECC containing different concentrations of TiO_2_ nanomaterial was ascertained. Following the grinding of ECC samples into a powder, 10-g powder were mixed in 100 millilitres distilled water while being agitated for ten minutes. Using a pH metre, the pH of the solutions was subsequently determined. Additionally, cube-shaped and bar (prism) specimens were fabricated and submerged in to a 10% sodium sulphate (Na_2_SO_4_) chemical, respectively, in order to assess the expansion and loss in compressive strength. The variation in sample dimension and compressive strength were documented monthly. Additionally, the concrete samples having cylindrical disc design with dimension of 100 × 50 mm were wet-cured initially for a duration of 28 days in order to conduct the RCPT test. AASHTO (T277-15)^56^ as well as ASTM (C1202-19)^57^ were utilised to conduct Rapid chloride permeability analysis. Before proceeding, quick setting epoxy were utilised to coat the concrete sections. Following the curing of the sealant coating, the cylindrical sample was subjected to the vacuum chamber for a duration of 3 h minimum, or till it ceased to exhibit excessive stickiness upon contact. Following this, the cylinder was inserted into the testing device containing 2 different cells that interacted with the 2 uncoated surfaces of the disc. The anode of the cell is composed of a 3% of weight of NaCl mixture in distilled water. The cathode of the cell is a 0.3N NaOH content in distilled water. Subsequently, the observing equipment was linked to a constant 60-V direct current (DC) power source for a period of 06 h. Throughout this time, the researchers measured the cumulative electric charge flowing through the disc specimen in coulombs at 30-min intervals. The overall charge denotes the amount of chloride ions that entered composite sample, as observed by the investigators^58^. Additionally, to assess the water absorption ability of ECC containing TiO_2_, three cubes from every batch were subjected to a designated process. In the beginning, these cubes were subjected to oven drying for 24 h at 105 °C to determine their original weight, which served as the baseline for evaluation. Thereafter, all specimens were immersed in water for 24 h to attain saturation. Thereafter, the ultimate mass of the samples was recorded as the dry weight of the saturated surface. The percentage of weight loss indicated the extent of water absorption by the specimens^59^. These samples were carefully dried at 105 °C to prevent any morphological modifications in the TiO₂ samples that may result from elevated temperatures, hence assuring precise water absorption evaluations^60^. Additionally, an additional set of three specimens was used to determine the apparent porosity by following ASTM C948-81^61^ procedure. Equation (1) was used to determine the apparent porosity (AP), yielding valuable information into the material’s pore geometry and its water retention capability.

1 $$Apparent Porosity = \left| {\frac{{W_{i} - W_{d} }}{{W_{i} - W_{s} }}} \right| \times 100\%$$

where Wi = Weight of the specimen during a 48-h immersion in water. Wd = Weight of the sample after moisture removal by exposure to an oven at 105 °C for 24 h. Ws = Weight of the specimen when suspended in water.

## Results and discussions

### Compressive strength (CS)

**Fig. 1:**
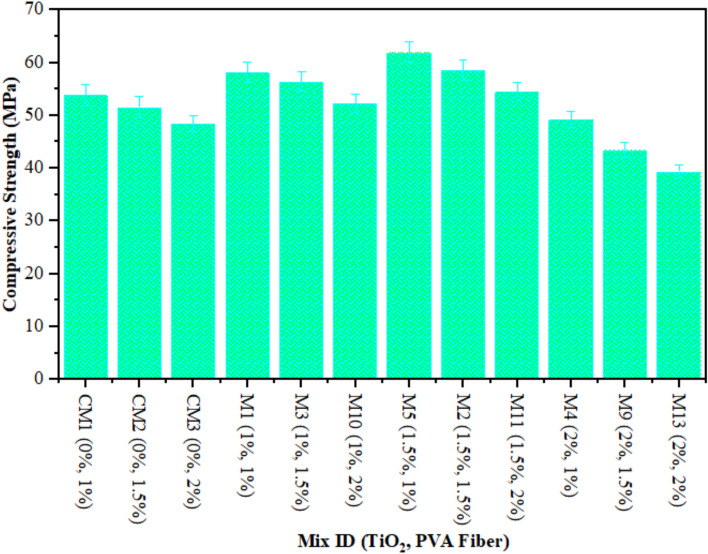
Compressive Strength of ECC containing TiO_2_.

The CS of composites containing 1% to 2% of PVA fiber by fraction of volume and different proportions of TiO_2_ nanoparticles after 28 days is illustrated in Fig. 1. The highest calculated strength of ECC combined with 1% of PVA fiber was noted by 62 MPa at 1.50% TiO_2_ as nanomaterial, while the lowest strength of composites contained 2% of PVA fiber was found by 39.40 MPa at 2% TiO_2_ as nanoparticle. There is argument regarding the optimal compressive strength achieved by adding 1.50% TiO_2_ to composites. Research also suggests that the strength of the mixture gradually reduces with subsequent additions of TiO_2_ to the ECC. The following factors optimise the strength of ECC when combined with 1.5% TiO_2_ as nanoparticle. (i) TiO_2_'s role: The formidable mechanical characteristics of TiO_2_ make a substantial contribution to the reinforcement of the cement matrix. The wrinkly TiO_2_ interconnecting multiple phases within the cement matrix may result in an increase in fracture surface hardness. (ii) The influence of pore-filling: The nanoscale nature of TiO_2_ facilitates its frequent and effortless infiltration into the cementitious matrix’s pore spaces, leading to enhanced densification. This further enhances the mechanical properties of the densified matrix. Consequently, TiO_2_ significantly contributes to filling the ECC mixture with mechanical properties. Furthermore, elevated levels of TiO_2_ incorporation can lead to TiO_2_ aggregation within the cementitious matrix. As a result, inadequate TiO_2_ dispersion reduces the CS of the ECC. It was detected that ECC with 1% PVA fiber and different amounts of TiO_2_ had higher CS after each curing time than the ECC mixture with 1.5% PVA fiber and different amounts of TiO_2_. According to the results, an ECC mix with 1.5% PVA fiber has a higher CS than one with 2% PVA fiber and unknown amounts of TiO_2_. Furthermore, ECC aggregated with 1% PVA fiber and different amounts of TiO_2_ has higher CS than ECC mixtures with 1.5% and 2% PVA fiber and several amounts of TiO_2_. The study shows that the CS goes down as the percentage of PVA fiber in composites with numerous contents of TiO_2_ rises. This reduction in the ECC mixture’s CS is the result of increasing the amount of PVA fiber, which generates more spaces, consequently reducing its CS. Moreover, the clustering behaviour of the fibers, which creates vulnerability areas within the mixture where the cementitious matrix is in short supply, is responsible for the observed strength reduction at elevated fiber concentrations. Numerous studies have proved that flocculated TiO_2_ significantly less effectively develop the mechanical properties of matrix than uniformly dispersed TiO_2_^62^. In a similar fashion, Sorathiya et al.^62^ increased the CS of cement by 22.6% during the curing period of 28 days by substituting 1% of the cement with TiO_2_. The research by Rawat et al.^52^, Baikerikar et al.^63^ and Orakzai^64^ is in line with these findings.

### Tensile strength (TS)

**Fig. 2:**
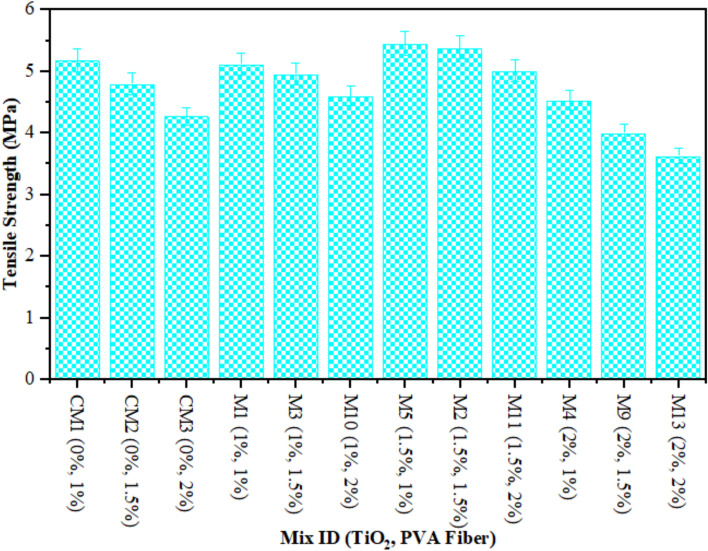
Tensile Strength of ECC containing TiO_2_.

The TS of composites containing 1–2% of PVA fiber by fraction of volume and different proportions of TiO_2_ nanoparticles after 28 days is illustrated in Fig. 2. The highest calculated strength of ECC combined with 1% of PVA fiber was noted by 5.46 MPa at 1.50% TiO_2_ as nanomaterial, while the lowest strength of composites contained 2% of PVA fiber was found by 3.625 MPa at 2% TiO_2_ as nanoparticle. There is argument regarding the optimal TS achieved by adding 1.50% TiO_2_ to composites. This enhancement in TS is due to pore filling influence of TiO_2_ at nanoscale in ECC, leading to enhanced densification. This further enhances the mechanical properties of the densified matrix. Furthermore, elevated levels of TiO_2_ incorporation can lead to TiO_2_ aggregation within the cementitious matrix. As a result, inadequate TiO_2_ dispersion reduces the TS of the ECC. It was detected that ECC with 1% PVA fiber and different amounts of TiO_2_ had higher TS after each curing time than the ECC with 1.5% PVA fiber and different amounts of TiO_2_. According to the results, an ECC mix with 1.5% PVA fiber has a higher TS than mixture combined with 2% PVA fiber and various amounts of TiO_2_. Furthermore, ECC aggregated with 1% PVA fiber and different amounts of TiO_2_ has higher TS than ECC mixtures with 1.5% and 2% PVA fiber and numerous amounts of TiO_2_. The study shows that the TS goes down as the percentage of PVA fiber in ECC composites with numerous contents of TiO_2_ rises. This reduction in the TS of ECC is the result of increasing the amount of PVA fiber, which generates more spaces owing to the balling effect and inadequate bonding to ECC with the addition of 2% PVA fiber to ECC^65^. The reduced density of ECC at elevated fiber levels may contribute to the decline in TS during cracking. Numerous studies have proved that flocculated TiO_2_ significantly less effectively develop the mechanical assets of matrix than uniformly dispersed TiO_2_^62^. Researchers^64^ conducted similar findings.

### Weight loss analysis

**Fig. 3:**
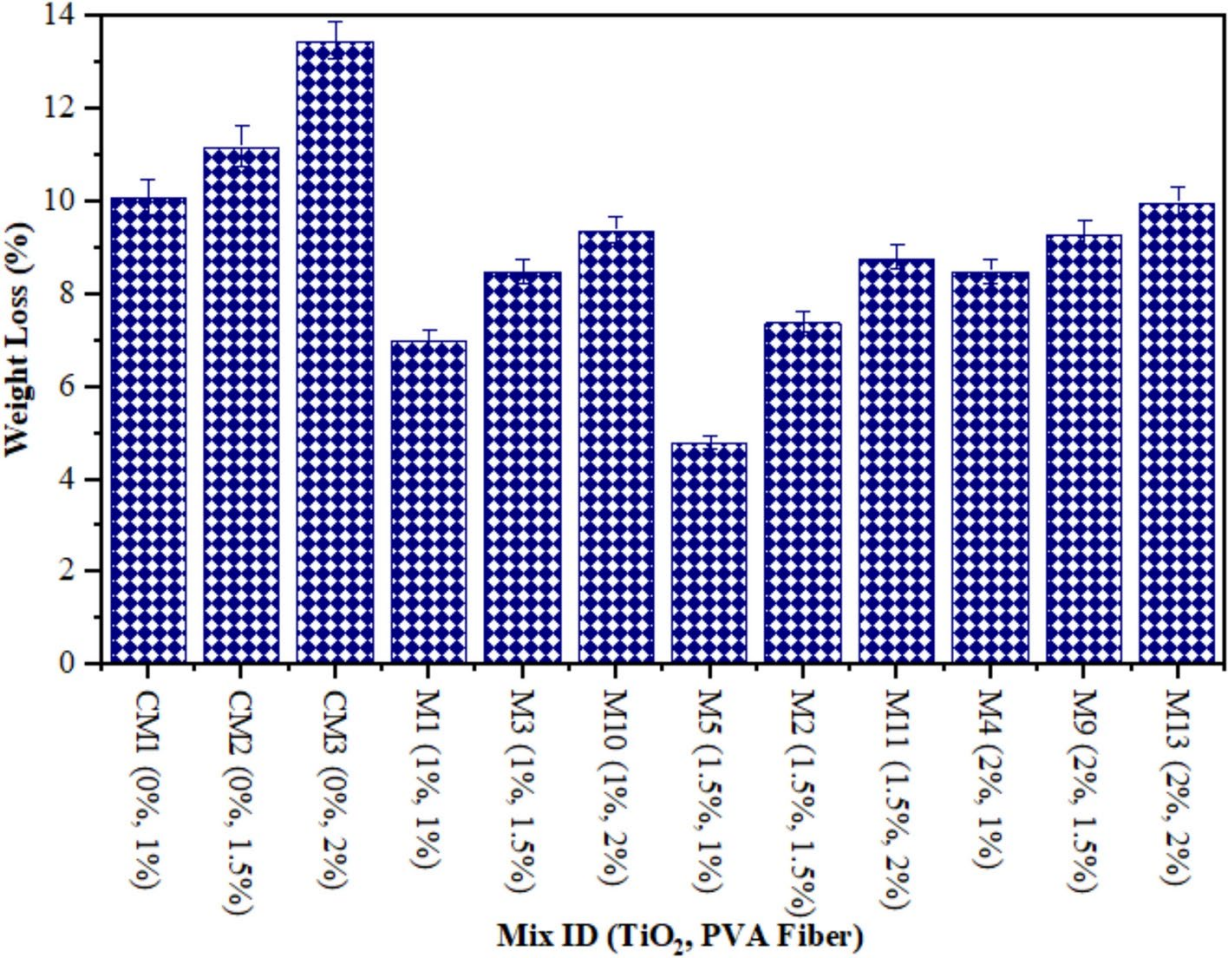
Weight Loss of ECC containing TiO_2_ due to Acid Attack.

Cubic specimens composed of TiO_2_-ECC were utilised to determine the weight loss caused by acid attacks. Figure 3 illustrates the results of a 1-month assessment of the weight loss of each specimen. Conversely, the TiO_2_-ECC integrated with 2% PVA fiber experienced a maximum weight loss of 10% at 2% TiO_2_, while the TiO_2_-ECC reinforced with 1% PVA fiber consistently showed a minimum weight loss of 7% at 1% TiO_2_ as nanoparticles after 28 days. Research shows that when TiO_2_-ECC incorporates 1% PVA fiber by volume fraction, acid attack causes the least amount of weight loss, compared to when TiO_2_-ECC incorporates 1.5% and 2% PVA fiber during production. In addition, the weight loss of mixtures M2, M4, M7, M8, and M13 was significantly greater than that of the other proportions. Due to its alkaline nature, TiO_2_-ECC is vulnerable to acidic attacks. The reaction between acid and calcareous substances produces calcium salts. The salts decrease the cohesiveness of the cement matrix and the density of the ECC. In addition, the C–S–H solution combine with sulfuric acid, resulting in a brittle gel and a reduction in the strength of TiO_2_-based composites. Besides, the presence of disintegrating calcium salts, namely calcium sulphate, leads to the formation of reaction products such as gypsum and ettringite. The occurrence of these reaction outcomes caused ECC’s density enhancement, thereby enhancing the reaction of hydration in the ECC mix. The Ettringite process and gypsum further reduced the porosity in ECC. Moreover, ECC experiences a marginal increase in mass while exposed to an acidic solution^66^. Equations (2) and (3) mathematically describe the reaction between ECC and a sulfuric acid solution^67^.

2 $${\text{Ca}}\left( {{\text{OH}}} \right)_{2} + {\text{H}}_{2} {\text{SO}}_{4} \to {\text{CaSO}}_{4} *2{\text{H}}_{2} {\text{O}}$$

3 $$3{\text{CaO}}*2{\text{SiO}}_{2} *3{\text{H}}_{2} {\text{O}} + {\text{H}}_{2} {\text{SO}}_{4} \to {\text{CaSO}}_{4} *2{\text{H}}_{2} {\text{O}} + {\text{Si}}\left( {{\text{OH}}} \right)_{4}$$

The results obtained from this experiment can be ascribed to the examination of the fluctuations in the concentrations parameters (PVA and TiO_2_). The microcracking of the specimens caused by the increased PVA particle content in the ECC enabled acidic media to permeate more deeply. Conversely, the ECC blended with a reduced percentage of PVA mix exhibited a diminished occurrence of fractures and a more difficult time separating the constituent elements, thereby leading to a reduced extent of weight loss as a result of deterioration. In addition, the TiO_2_ in the composition enhanced the ECC’s resistance to degradation under extreme conditions, such as acidic environments. Due to ECC degradation, compositions containing less TiO_2_ experienced greater proportions of weight loss, whereas samples containing more TiO_2_ exhibited more durability. By comparing mixtures containing 1% PVA with varying concentrations of TiO_2_, it was determined that mixtures with a higher TiO_2_ content exhibited superior weight loss results.

### Compressive strength due to acid attack

**Fig. 4:**
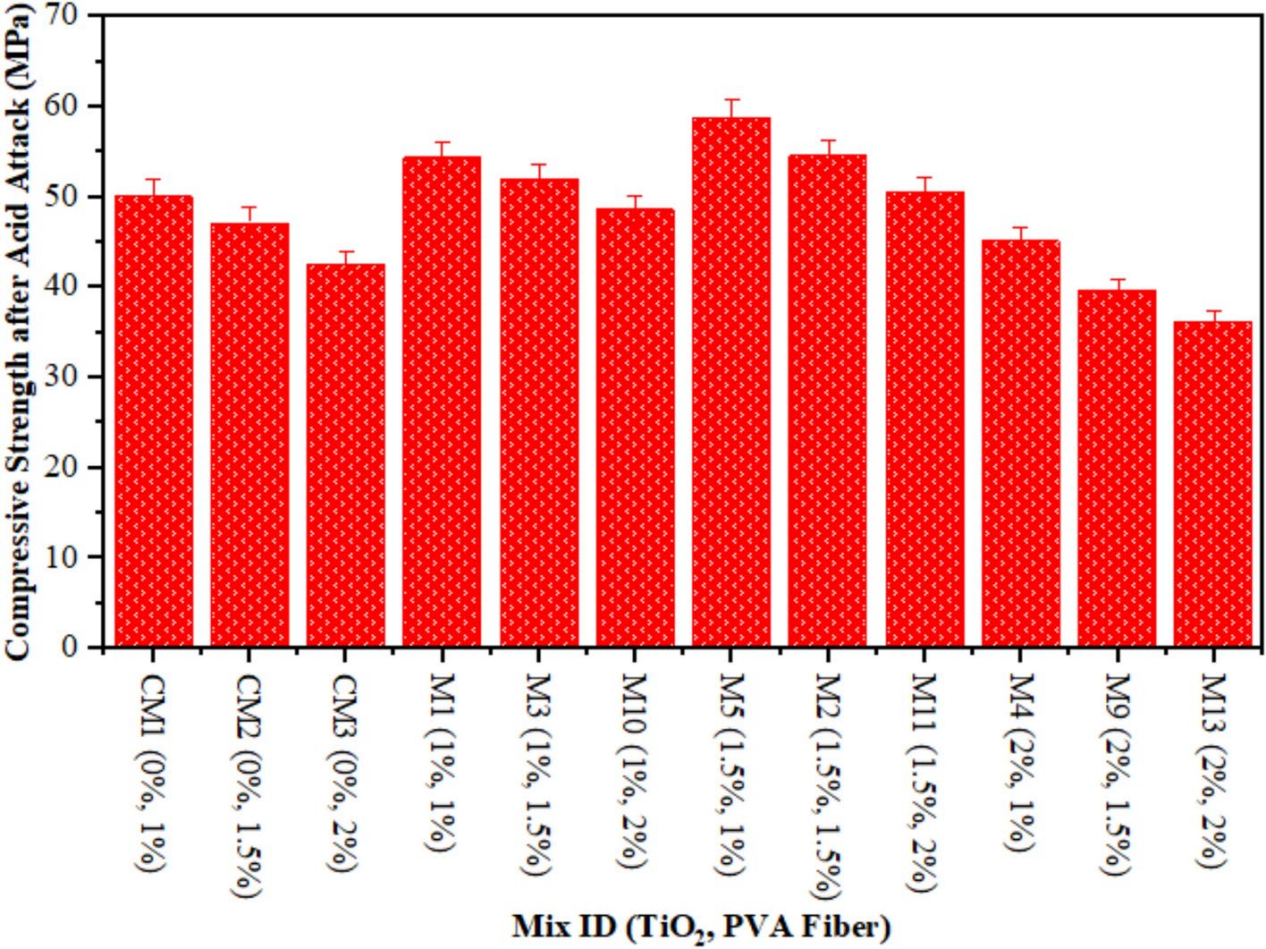
Compressive Strength of ECC containing TiO_2_ due to Acid Attack.

**Fig. 5:**
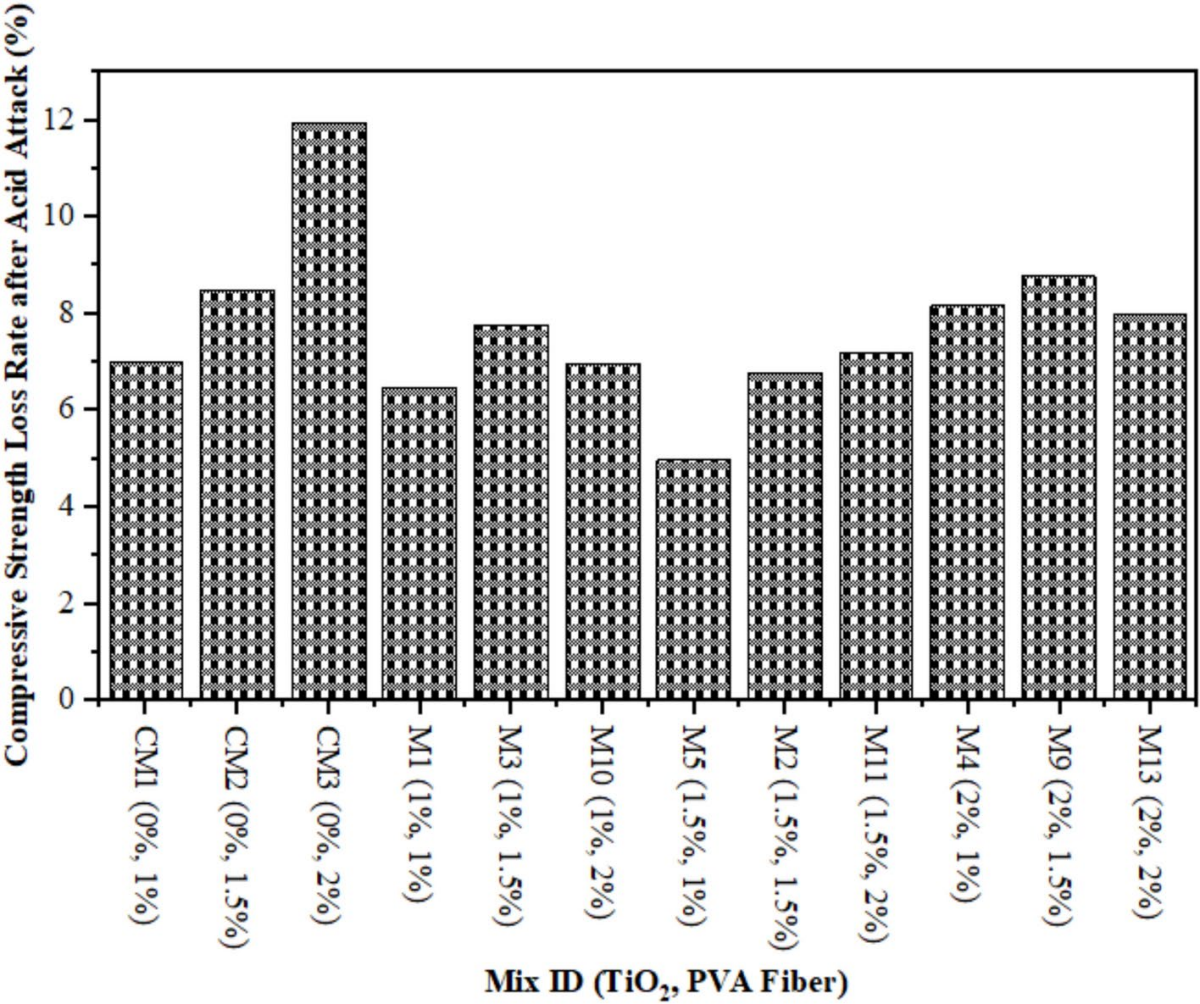
CS Loss Rate in Percentages after Acid Attack.

Figure 4 illustrates the outcomes of a CS on ECC samples that underwent an acidic attack with a duration of 28 days. It is very imperative to assess the strength and properties of the all prepared TiO_2_-ECC samples after an acid attack to find out how much damage was done and how fast the samples are breaking down. Twenty-eight days after being submerged in a 10% sulfuric acid solution, samples were evaluated for CS. Figure 5 illustrates the CS of CM and samples exposed to acid attack, along with the corresponding fraction reduction in CS on 28 days. A typical control sample experienced a reduction in CS of up to 12%. After 28 days of incorporating 1.50% TiO_2_ nanoparticles, the acid attack reduces the CS degradation of composites reinforced with 1% PVA to a minimum of 6.50%. The decrease in CS loss was observed in the TiO_2_-ECC sample, which comprises 1.50% TiO_2_ by weight of cement, after a 28-day acid attack. Adding TiO_2_ as a nanoparticle enhance to the ECC mixture also makes it less likely that the CS will decrease when it is attacked by acid, compared to CM. Furthermore, the addition of 1.50% TiO_2_ to the ECC mixture increased hydration in all mechanical properties. Another thing is that the TiO_2_-ECC sample showed better resistance to degradation and compressive forces after being attacked by acid for 28 days. The samples of ECC contained with 1.50 percent TiO_2_ demonstrate a 6.50 percent reduction in CS loss. The integration of TiO_2_ into ECC significantly reduced degradation and enhanced sulfuric acid resistance. Furthermore, researchers attribute the decrease in CS in acid-attacked concrete samples to enhance in porosity and micro-voids^68^. Anatomically, Aiken et al.^69^ and Chintalapudi and Pannem^70^ documented comparable outcomes.

### pH assessment

**Fig. 6:**
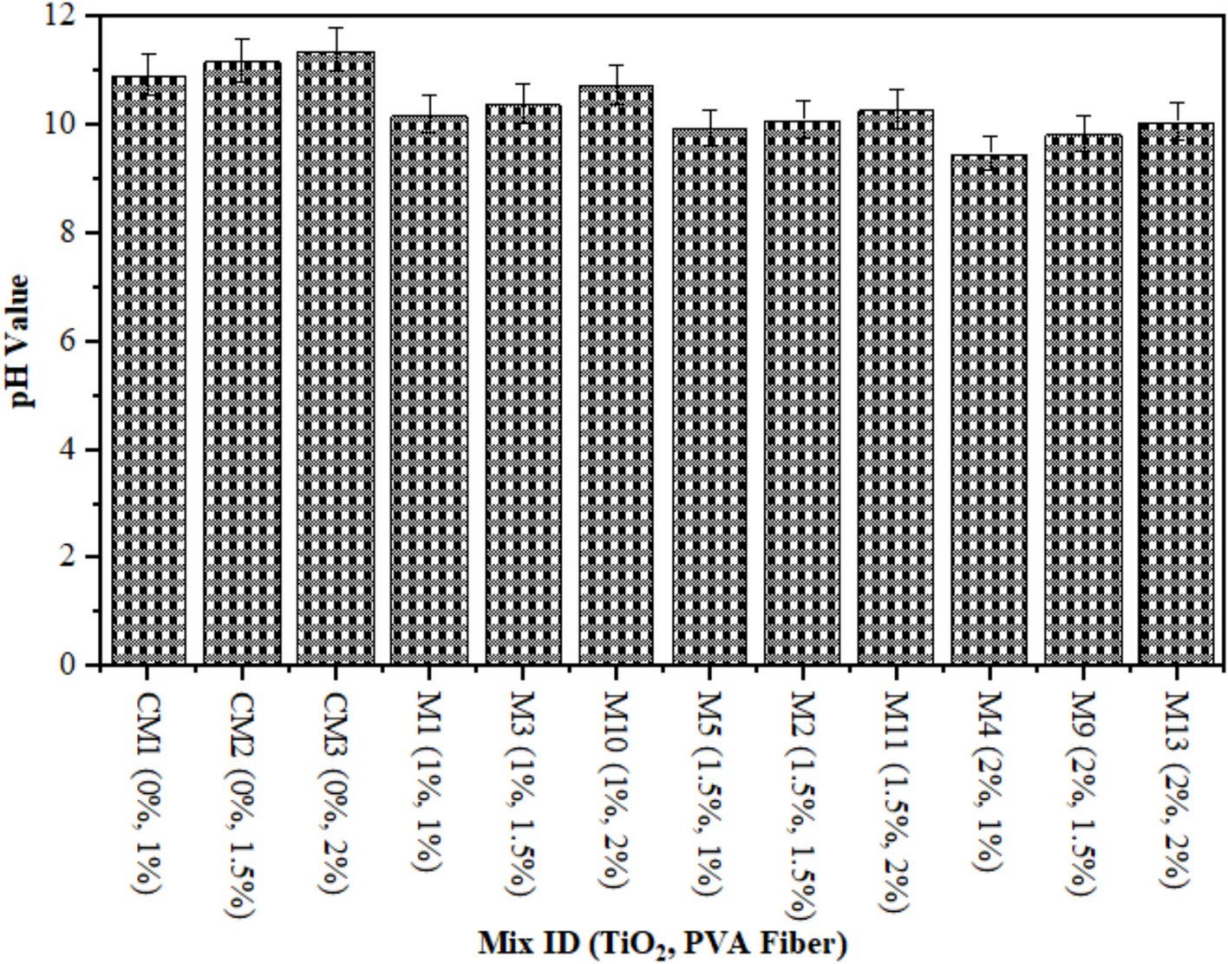
pH reading of Composites containing TiO_2_.

The assessment of acidic attacks that could cause significant damage to reinforced concrete requires ECC’s power of hydrogen (pH) value measurement. This is due to the fact that embedded steel exhibits sustained durability exclusively at pH levels of 9 or higher. As shown in Fig. 6, concrete samples comprised of ECC containing 0–2% TiO_2_ nanoparticles were monitored for pH value after 28 days. At 1%, 1.5%, and 2% TiO_2_ as nanomaterial, the pH value of matrix contains with 1% PVA fiber was 10.20, 9.95, and 9.48, respectively, which was consistently lower than CM at 28 days. In a similar way, the pH result for ECC reinforced with 1.5% PVA fiber and 1%, 1.50%, and 2% TiO_2_ nanoparticles was found by 10.40, 10.10, and 9.85 which is consistently lower than CM at 28 days correspondingly. Research has demonstrated that when 1% PVA fiber is used to accumulate TiO_2_ nanoparticles in ECC, the resulting pH value is lower than 1.5% PVA fiber utilized in ECC with the addition of various amounts of TiO_2_ nanoparticles. Furthermore, the pH values of ECC incorporated with 2% PVA fiber recorded by 10.75, 10.30, and 10.08, when 1%, 1.50%, and 2% TiO_2_ were used as nanoparticles on 28 days respectively. After 28 days, the pH values of all mixtures consistently reduced in comparison to the CM. It has been determined that the addition of different concentrations of TiO_2_ to ECC results in a greater accumulation of PVA fibers, as the pH value increases due to the nanoparticles. However, it is worth noting that these all-pH values are lesser than CM. Furthermore, it is observed that the pH level of composites combined with varying percentages of PVA fiber is reduced as the concentration of TiO_2_ nanoparticles rises. The drop-in pH rate observed in composites comprised with different percentages of PVA fiber can be accredited to the refining of TiO_2_ at a nanoscale. This effect effectively covers the openings in ECC and protects it from acid attack and environmental degradation, both of which contribute to the pH reading reduction. Wan et al.^71^ reported that carbonation has the capability to decrease the pH reading from 13.20 to 8.0. It can be concluded that the carbonation is not only induces a reduction in pH but also liberates chloride ions. The collective impact of chloride ingress and carbonation speed up the rust rate of steel and decreases the life of structures. Kumari et al.^72^ demonstrated that as the proportion of nanomaterials in concrete replacement increased to 2%, there was an initial increase in pH followed by a gradual reduction. An analogous form of investigation was undertaken by Zeng et al.^73^.

### Rapid chloride penetration test (RCPT)

The objectives of this research examined the impact of nanoscale particle on the ECC’s water transport properties. The effects of water transport through the ECC samples combined with TiO_2_ were observed. Once chloride ions penetrate the ECC, it need to bind to various products from hydration. So, the overall chloride ions that get into ECC are made up of bound chloride ions and the amount of free chloride ions that are in the pores. Free chloride ions permeating rebar and passing through ECC pores initiate rusting. It is calculated chloride permeability in agreement with ASTM C1202^57^ using acid-soluble chloride^74^.

**Fig. 7:**
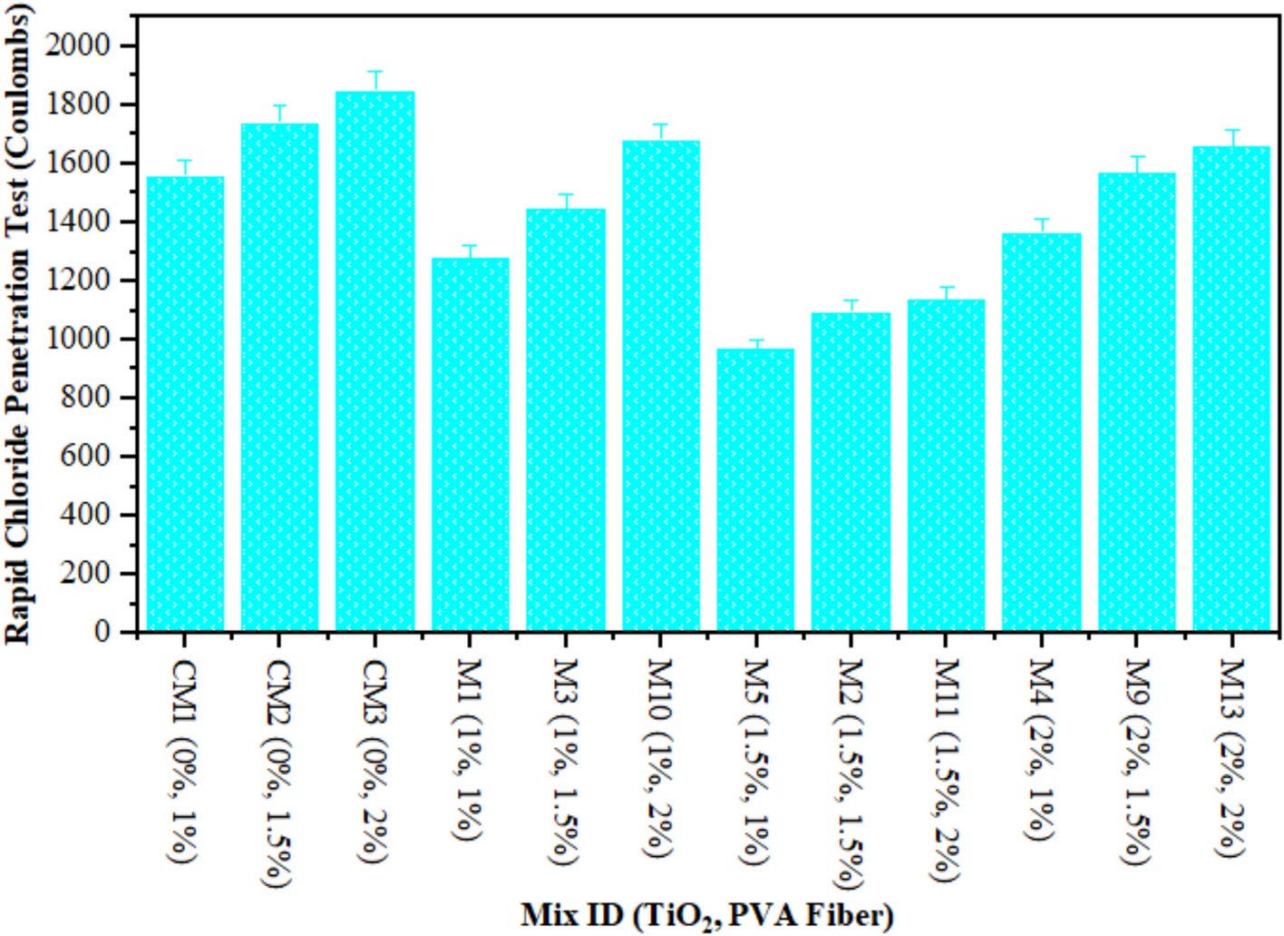
RCPT of ECC containing TiO_2_.

This investigation recorded the applied current discharge in 6 h as an indicator of chloride permeability. Figure 7 illustrates the RCPT of composites contained various content of TiO_2_ as nanoscale component at 28 days. However, the RCPT reading for CM and ECC containing TiO_2_ that adhere to ASTM C1202^57^ for low chloride permeability varied between 968 and 1850 coulombs. Moreover, an increased TiO_2_ concentration during ECC synthesis reduces chloride ion permeability. The RCPT readings of composites contained 1% PVA fiber were noted by 1278, 968, and 1365 Coulombs at 1%, 1.50%, and 2% TiO_2_ respectively, which is lower than CM. Similarly, the RCPT values of matrix mixed with 1.5% PVA fiber and 1%, 1.50%, and 2% TiO_2_ nanoparticles were always lower than those of the CM on 28 days. In addition, the RCPT of composites with 2% PVA fiber at level of 1%, 1.50%, and 2% dropped as compared to reference mixture. It has been realized that an increasing quantity of TiO_2_ in composites, which leads to a decrease in RCPT. The decrease in microporous interconnectivity and microstructural densification of ECC is responsible for the decline in RCPT values. The use of TiO_2_, as nanoparticles with nanoscale dimensions, to form a microstructure that was densely packed and compact, thereby demonstrating enhanced resistance to chloride penetration. To determine the RCPT, Khotbehsara et al.^74^ discussed the use of nanomaterials such as SnO_2_, ZrO_2_, and CaCO_3_ in self-compacting mortar. Nevertheless, beyond a certain threshold, the RCPT value decreased as the nanomaterial concentration increased. As previously mentioned, nanomaterials function as fillers to increase the structure’s density and fill in any gaps. Additionally, they facilitate the hydration process. This expedites the formation of a more developed pore structure, which reduces the amount of chloride ion transport. This study’s results suggest the incorporation of nanoparticles into cement mortars to enhance the resistance against infiltration of chloride ions. A portion of the chloride ions binds to the composites, which slows the diffusion rate. Prior research^75^ reported that the permeability of chloride of glass powder-containing concrete mixtures was lesser than CM. Miyandehi et al.^76^ documented a substantial reduction in the quantity of transmitted charge upon the introduction of copper oxide nanoparticles.

### Expansion of TiO_2_-ECC

**Fig. 8:**
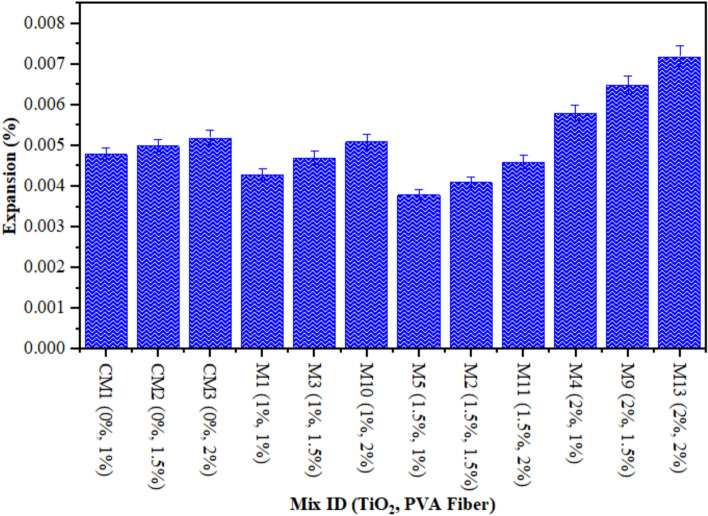
Expansion of ECC containing TiO_2_ due to Sulphate Attack.

Figure 8 illustrates the expansion resulting from the sulphate attack on the accumulation of ECC matrix with varying concentrations of TiO_2_ nanomaterials. Alternatively, it found the expansion of prism length prior and after their submergence in a 10% sodium sulphate solution over a 28-day period. The percentage increase was subsequently calculated. Based on the study outcomes, M5 (1.50% TiO_2_ and 1% PVA) had the lowest expansion of about 0.0038%. In contrast, M13 (2% TiO_2_ and 2% PVA) displayed the most substantial expansion, with an expansion percentage of 0.0072%. Furthermore, the samples denoted as M4 (2% TiO_2_ and 1% PVA fiber), and M9 (2% TiO_2_ and 2% PVA fiber) demonstrated the most substantial increment in ECC production after a duration of 28 days. Analogous patterns were noted in^77^. The presence of 1%, 1.5%, and 2% fiber, along with TiO_2_ in the range of 1% to 2%, may account for the observed results. Coats containing a higher concentration of PVA expand more rapidly due to the micropores that surround the sample’s surface, facilitating improved absorption of immersed media. As the extent of PVA increases, there are more micro-voids in the mixture, resulting in a higher degree of expansion. The study concluded that concrete mixtures containing PVA at a concentration exceeding 1% expanded more than the control sample. Furthermore, the integration of TiO_2_ into the concrete lead to in a reduction in expansion due to the concrete’s resistance to infiltration caused by sulphate attack. During the dissolution and establishment phases of ECC containing TiO_2_, a reduction in free water led to a rise in electrical resistivity. As the hydration process increases, solid by-products build up and porosity decreases. This makes it harder for ions to move through the TiO_2_ paste-containing ECC, which raises the resistance. A higher concentration of TiO_2_ in concrete may mitigate the effects of expansion caused by sulphate suspension infiltration. Additionally, the formation of ettringite-filled fissures and fractures on the surface of the cement composite contributed to the increase in length change^78,79^. Furthermore, the formation of ettringites, which occupy a more area, contributes to the expansion and fracture characteristics of concrete^80^. This is in line with prior study that has examined the effects of sulphate on concrete^66,81^.

### Compressive strength of TiO_2_-ECC due to sulphate attack (SA)

**Fig. 9:**
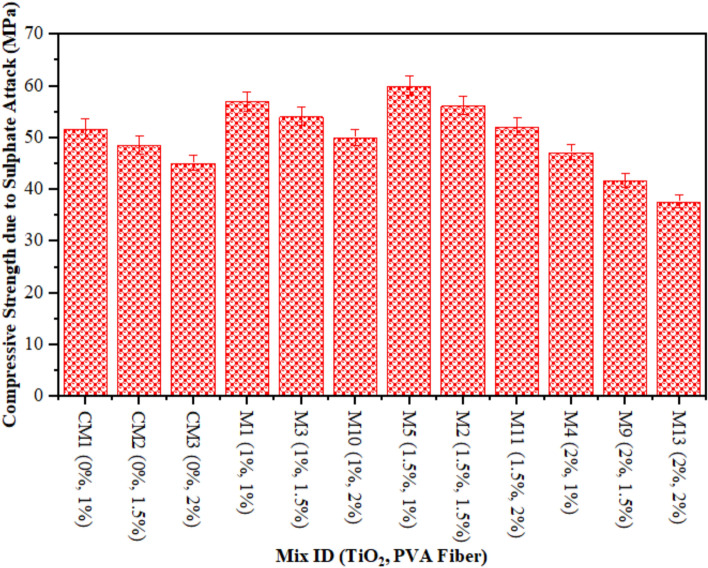
CS of ECC containing TiO_2_ due to SA.

**Fig. 10:**
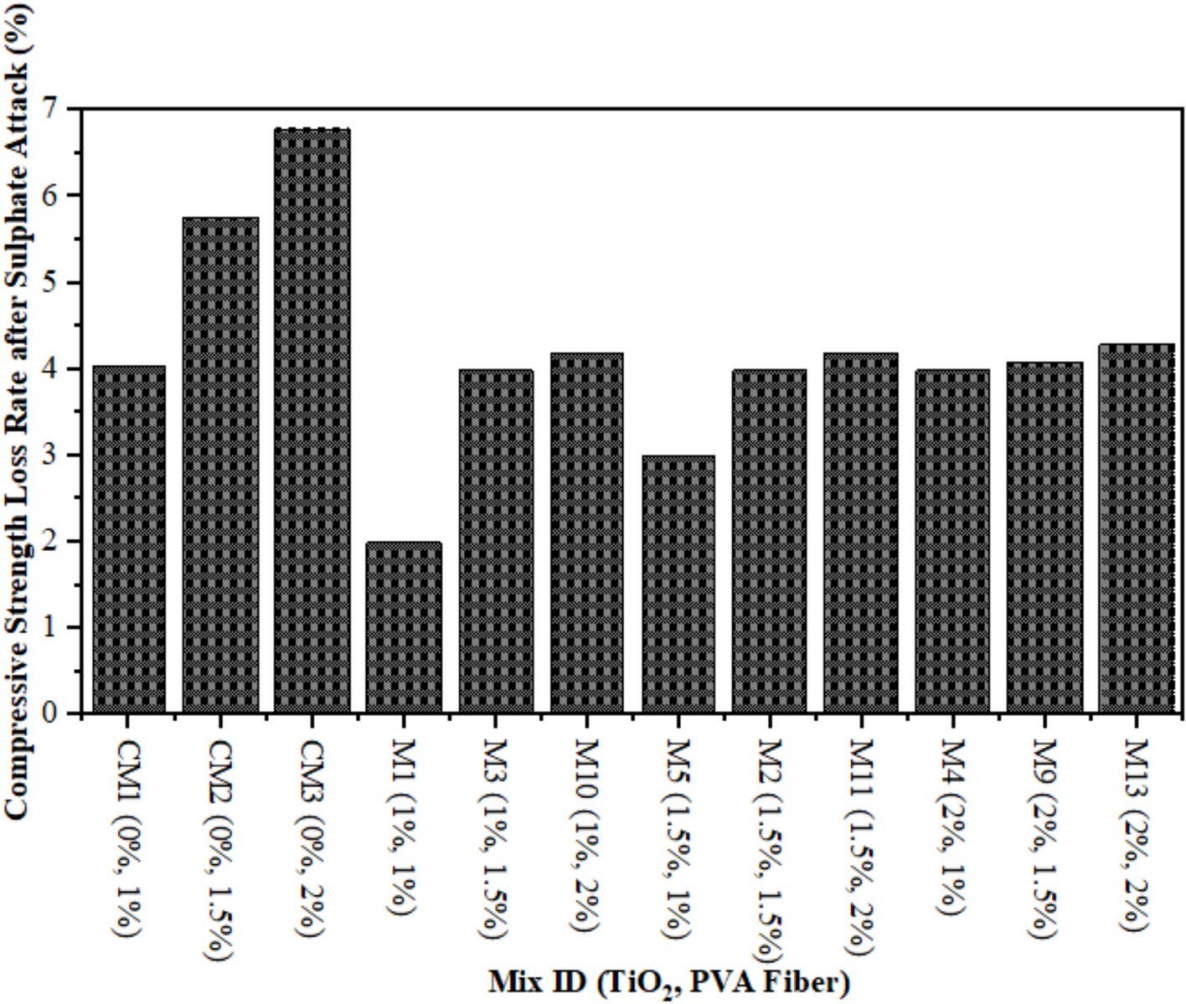
CS Loss Rate in Percentages after SA.

A comparison is made between the CS reduction due to SA in composites and CS without SA specimens that underwent 28 days of water curing. Figure 9 illustrates the result. The reduction is relatively minor, amounting to a meagre 1.50% TiO_2_ as nanoparticles, when compared to CM. However, after a period of 28 days, the control mix experiences a substantial decline of 3.23%. After 28 days, the CS of an ECC mixture containing 1% PVA fiber consistently decreases by 2%, 3%, and 4% at 1%, 1.50%, and 2%, respectively as shown in Fig. 10. Similarly, at 28 days, the strength of ECC incorporated with 1.5% PVA decreases by 4%, 4.05%, and 4.10%, respectively, when 1%, 1.50%, and 2% TiO_2_ nanoparticles are added to ECC. An identical pattern is observed in TiO_2_-ECC that is fortified with 2% PVA fiber. The results indicate that the addition of 1.50% TiO_2_ could potentially offer substantial protection against sulphate attack. In addition, ECC performance frequently degrades, and the immersion time is prolonged when exposed to a chemical assault because of physical degradation and unintended chemical degradation of composites. As illustrated in Fig. 10, the introduction of TiO_2_ resulted in an augmentation of resistance against sulphate attack. The CS of water-cured concrete mixtures was compared to the results of CS tests conducted 28 days after sulphate assaults on the mixtures. It was demonstrated that the magnitude of the loss in CS increased in CM as compared to ECC combined with the TiO_2_. Moreover, the sulphate attack induces the liberation of calcium constituents from C–S–H gels, ensuing in a decrease in the rigidity of the composites and subsequent degradation of the samples^82^. Honglei et al.^83^ identified two strata of gypsum accompanied by minute amounts of ettringite and monosulfate. Sulphate attack is characterised by a reduction in adhesion and strength, in contrast to the formation of fissures and thickening^76^. The sulphate reactions altered each of the compounds, which led to a reduction in CS. These outcomes are analogous to these prior investigations^84,85^.

### Apparent porosity (AP)

**Fig. 11:**
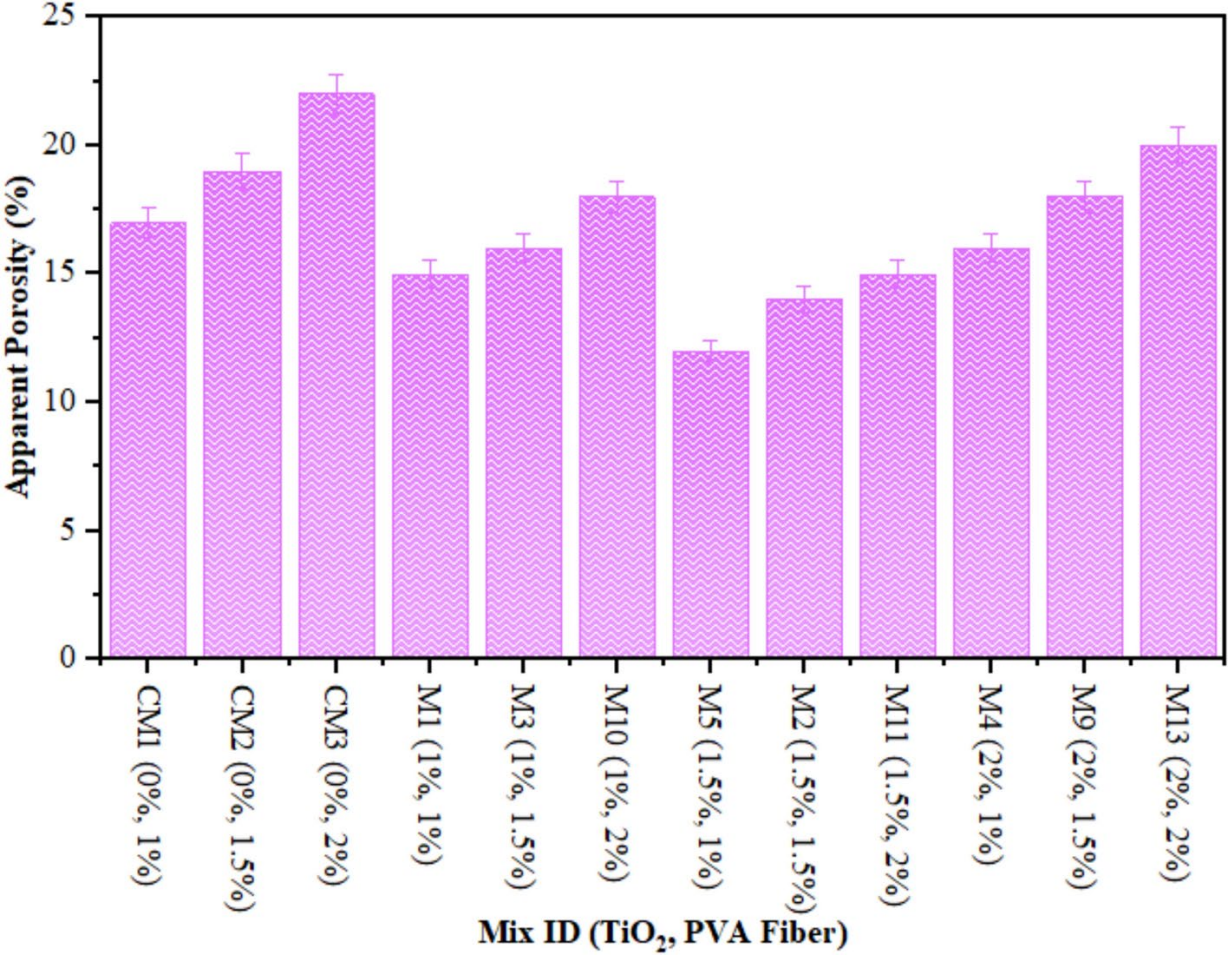
Apparent Porosity of ECC containing TiO_2_.

Figure 11 illustrates the apparent porosity of ECC, which includes varying amounts of PVA fiber and TiO_2_ as a nanomaterial, after a 28-day duration. Notably, the porosity of all mixes including TiO_2_ was inferior to that of the control mixture. Despite this, adding PVA fiber made the concrete more porous. This was because the higher volume fraction of PVA fiber caused more air to be trapped in the ECC mixture. This characteristic led to an increase in capillary water levels, which in turn increased the porosity as the concentration of PVA fiber increased. The inclusion of TiO_2_ at nanoscale in composite caused in a considerable reduction in porosity across all PVA fiber levels. The reduction in porosity may be attributed to TiO_2_ nanoparticles functioning as efficient fillers. At smaller dosages, TiO₂ nanoparticles exhibit superior dispersion, efficiently occupying voids and refining the pore morphology. Nevertheless, as the amount rises to 2%, agglomeration becomes more evident. These agglomerates produce weak zones and interfacial flaws, resulting in enhanced porosity. Mohammadi et al.^86^ conducted research that demonstrated a decrease in overall porosity upon the incorporation of titanium dioxide into calcium phosphate cement. These discoveries align with findings from prior studies by Nazari and Riahi, which noted a reduction in porosity when PC was partly replaced by TiO_2_ at varying concentrations. Their findings indicated that a reduction in porosity was attained with a 3% dosage of TiO_2_, resulting in decreases of 5.67% in porosity. Similar findings were reported by other scholars^60,87^.

**Fig. 12:**
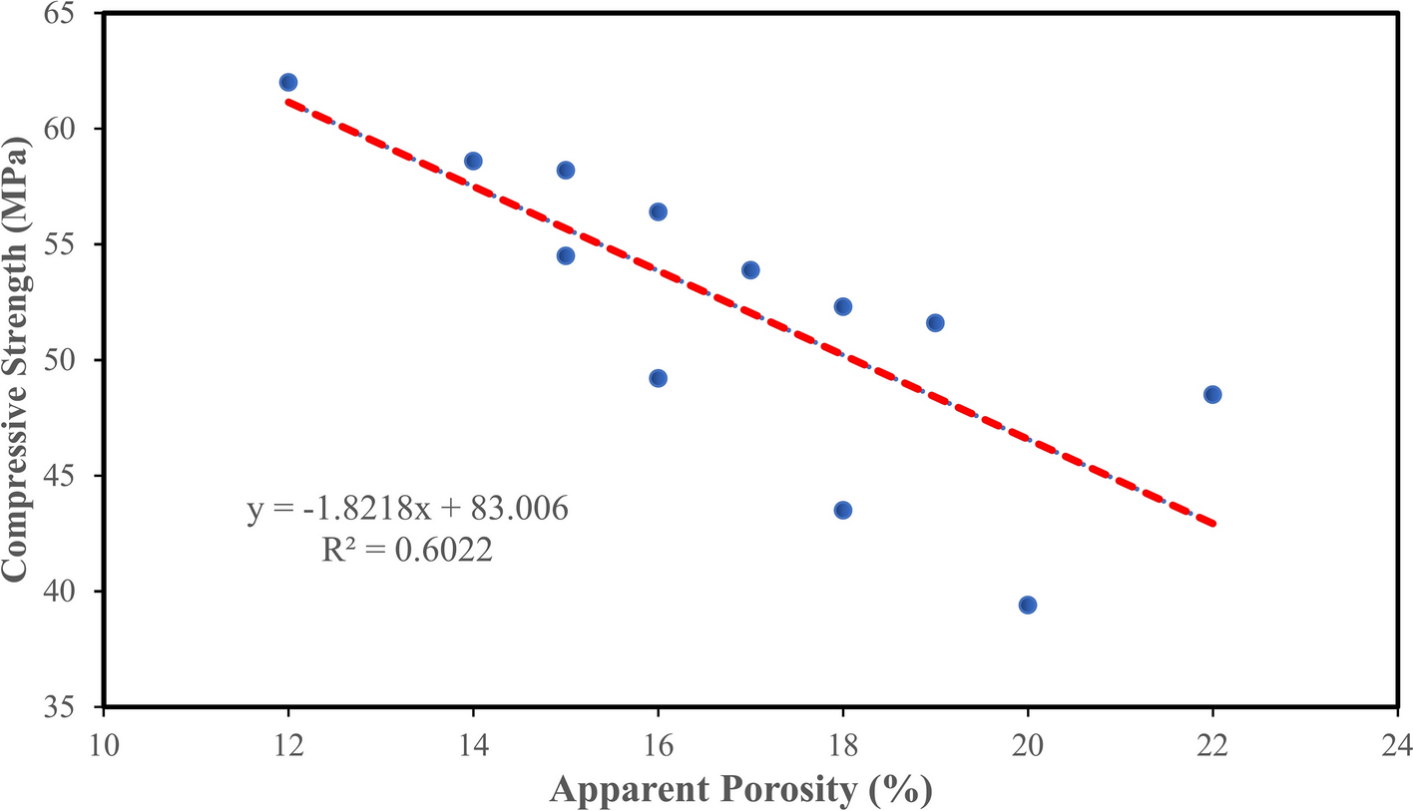
Relationship between AP and CS.

**Fig. 13:**
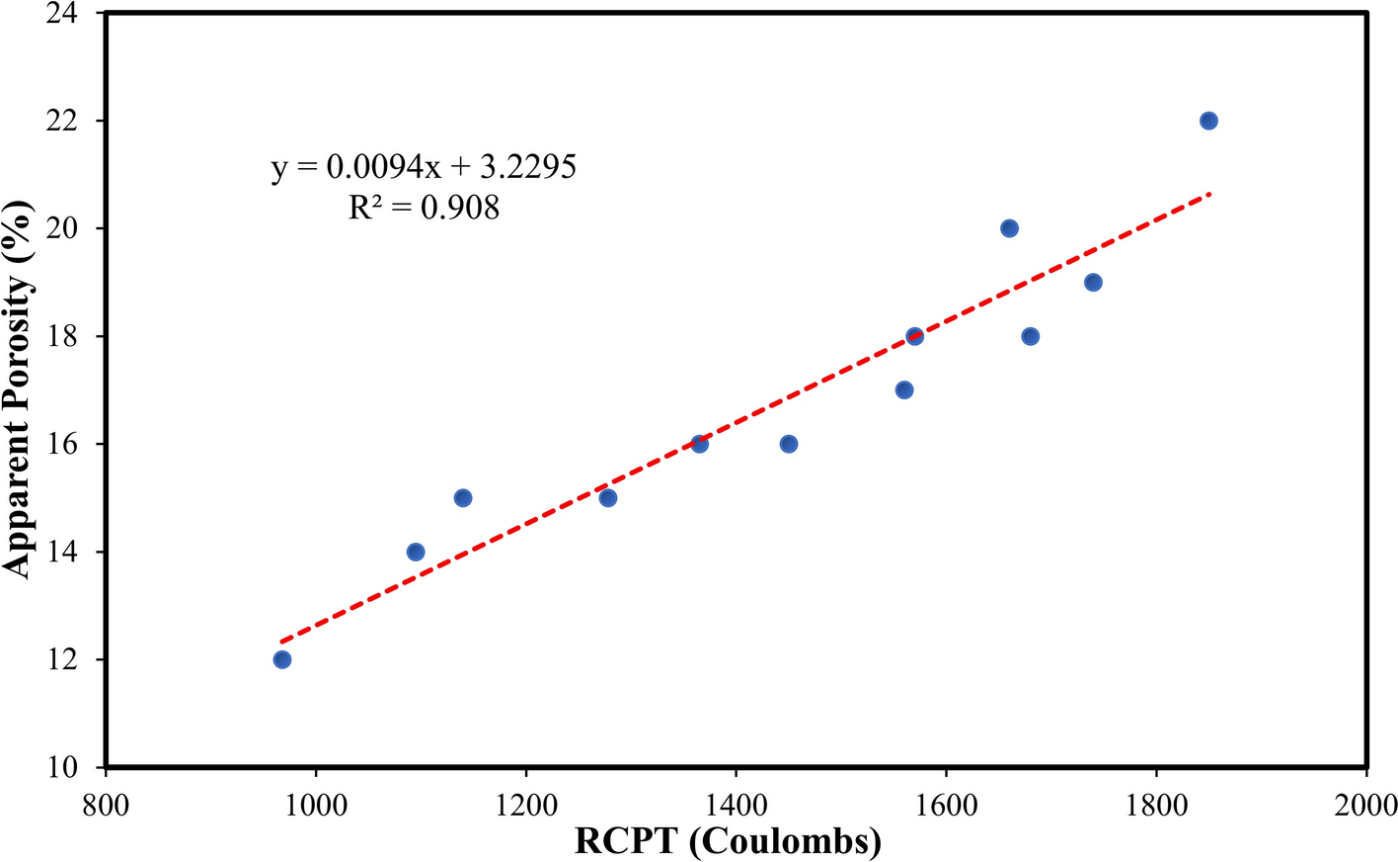
Correlations between the durability indicator (RCPT) and AP.

The relationship between apparent porosity and compressive strength is illustrated in Fig. 12. Figure 12 illustrates a strong connection between apparent porosity and compressive strength after 28 days. The equations in Fig. 12 will aid in forecasting the compressive strength or apparent porosity whenever one of these characteristics is identified. The experimental results demonstrated a favourable relationship regarding the regression curve outcomes. The test findings assessed the strength and porosity performance of concrete incorporating TiO₂ and PVA fiber in varying amounts inside composites. Similarly, the linear relationship between apparent porosity and durability indicator (RCPT) is shown in Fig. 13. Figure 13 illustrates a robust connection between apparent porosity and durability indicator (RCPT) on 28 days. The equations in Fig. 13 will help in predicting the apparent porosity or durability indicator (RCPT) if one of them is identified. The outcomes evaluated the apparent porosity and durability indicator (RCPT) performance of concrete containing TiO₂ and PVA fiber in different proportions in composites.

### Mercury intrusion porosimetry (MIP)

**Fig. 14:**
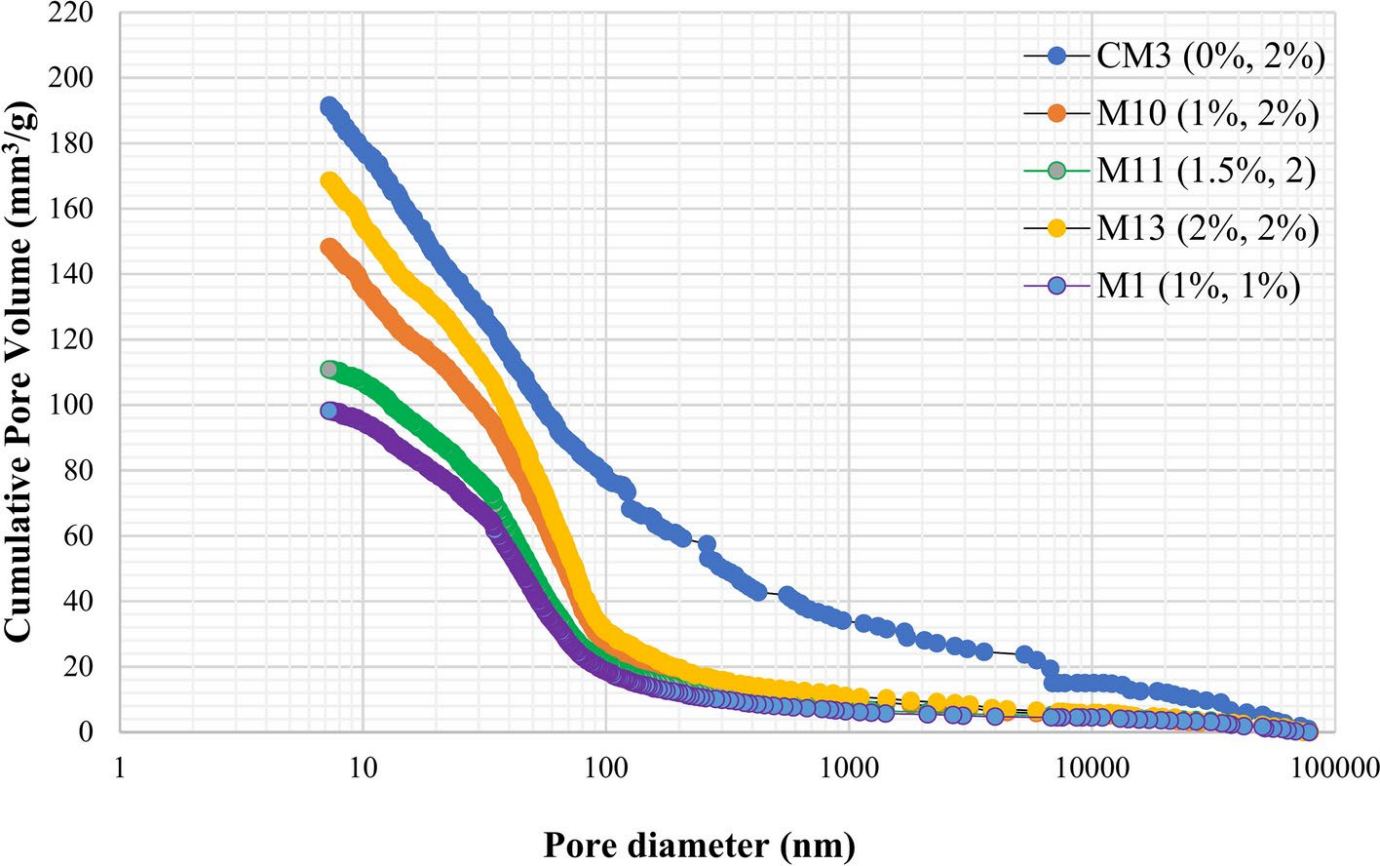
Pore Distribution of Composites blended with TiO_2_.

The MIP assessment was performed on a particular set of samples to examine the features of the pores and analyse the effects of input variables on the structure and distribution of the pores. The most direct method for understanding pore size distribution is to analyse the pore size distribution curve (PSDC), as illustrated in Fig. 14. Moreover, pore distribution is essential as it affects the permeability of the microstructure. The pore size distribution curve for the control sample, as illustrated in Fig. 14, reveals a total pore volume of 191.55 mm^3^/g. Moreover, the composite has a uniform distribution of pores of differing dimensions. However, the incorporation of 1.5% TiO₂ and 2% PVA fibers resulted in a reduction of the entire pore volume to 110.80 mm^3^/g. Furthermore, TiO₂ resulted in a reduction of pore amount throughout the 100–1000 nm range, thereby increasing the proportion of smaller voids measuring less than 100 nm, as illustrated in Fig. 14. The findings demonstrate TiO₂'s ability to improve hydration and nanofiller functions, leading to pore refining. At smaller dosages, TiO₂ nanoparticles exhibit superior dispersion, efficiently occupying voids and refining the pore morphology. Nevertheless, as the amount rises to 2%, agglomeration becomes more evident. These agglomerates produce weak zones and interfacial flaws, resulting in enhanced porosity. Moreover, it is notable that the increase in PVA fiber concentration led to a proportional enhancement in the total pore volume. The results presented in Fig. 14 prove this observation, indicating that the sample composed of ECC with 1% PVA fiber and 1% TiO₂ demonstrated the entire pore volume of 98.20 mm^3^/g, whereas the specimen comprising 2% PVA fiber and 1% TiO₂ revealed an overall pore volume of 148.20 mm^3^/g. The outcomes correspond with those reported by Long et al.^88^, indicating that the incorporation of PVA fiber during the procedure of mixing significantly increases air content. This increase in air fraction ultimately results in heightened porosity and finally permeability of ECC. Therefore, the addition of TiO_2_ in composites which decreases the pore size, considerably reduces the pore connectivity and decreasing the permeability of composites.

## Optimization utilizing response surface methodology (RSM)

The RSM framework comprises a collection of mathematical and statistical methodologies utilised to construct analytic models^89–91^. These methodologies rely on a response, or output, that is linked to a multitude of independent input parameters. By utilising this methodology, one can assess the influence of every variable as well as the interrelation among them on the outcome^92^. In this investigation, prediction models and optimisation of the CS, RCPT, sulphate attack, and acid attack, as well as the quantities of TiO_2_ and PVA in the TiO_2_-ECC, were accomplished via RSM. The optimisations and design strategies were generated with the assistance of design expert software. The optimisation process comprises three fundamental stages: (1) conducting an experimental investigation that is statistically devised; (2) determining the values of the variables in a scientific approach; and (3) predicting the outcomes of the model and confirming its appropriateness^93,94^. The statistical approaches may suggest that the relationship between responses and independent factors is either linear or a higher-degree polynomial. The 1st order equation signifying the linear model is illustrated by Eq. (4). In Eq. (5), the polynomial equations are expressed. Response models are represented by the y sign; the coded values of the input factors are denoted as *xi* and *xj*; the linear and quadratic parameters are denoted as *i* and *j*; the intercept on the y-axis is denoted as βo; the number of independent factors utilised in approach is denoted by k; and the error in the produced model is denoted as:

4 $$y = \beta_{0} + \beta_{1} x_{2} + \beta_{2} x_{2} + \beta_{n} x_{n} + \epsilon$$

5 $$y = \beta_{0} + \mathop \sum \limits_{i = 1}^{k} \beta_{i} x_{i} + \mathop \sum \limits_{i = 1}^{k} \beta_{ii} x_{i}^{2} + \mathop \sum \limits_{j = 2}^{k} \mathop \sum \limits_{i = 1}^{j = 1} \beta_{ij} x_{i} x_{j} + \epsilon$$

Following the determination of the variables’ statistical significance with a p-value of 0.05, a variance analysis was performed. In determining the significance of model responses, the fundamental and interacting characteristics of the parameters with p-values below 0.05 were observed as crucial, while those with p-values exceeding 0.05 were considered inconsequential^95,96^. Prediction models only consider pertinent terms, except for those necessary for the model’s structure. The experimental parameters that were considered were TiO_2_ and PVA. In this investigation, the following variables were studied: expansion, weight loss, CS, CS resulting from sulphate attack, pH test, and RCPT. Thirteen random mixtures were generated by the software, with five duplicates for every response. The principal targets utilised by software to improve the response of the experiment to potential deviations are the five duplicates.

### Analysis of variance (ANOVA)

The primary aim of RSM is to construct models and conduct ANOVA on them. In contrast, the CS and durability properties of ECC mixtures containing varying proportions of PVA fiber and TiO_2_ nanoparticles (ranging from 1 to 2%) were investigated for the purpose of RSM modelling and optimisation. Furthermore, for calculating CS, TS, CS due to acid attack, RCPT, CS due to SA, and AP quadratic models were considered the most appropriate. On the other hand, for calculating expansion and weight loss response, linear models were regarded more suitable. Additionally, the encoding of each of these responses can be found in Eqs. (6) through (14). By defining equations in terms of coded components, the impact of altering parameter quantities can be predicted. By default, the lowest values of the components are indicated as − 1, whereas the maximum values are presented as + 1. By applying the corresponding weightings to the factor variables, the coded equations can be utilized to find the comparative importance of the parameters. The input factors A and B are TiO_2_ and PVA, respectively. Details of the ANOVA results is given in Table 3.

**Table 3:** ANOVA Results.

Response	Source	Sum of squares	Df	Mean square	F-value	*p*-value > F	Significance
Compressive strength	Model	620.43	5	124.09	510.58	< 0.0001	Yes
	A- TiO_2_	254.77	1	254.77	1048.32	< 0.0001	Yes
	B-PVA Fiber	97.59	1	97.59	401.57	< 0.0001	Yes
	AB	4.78	1	4.78	19.68	0.0030	Yes
	A^2^	218.48	1	218.48	899.01	< 0.0001	Yes
	B^2^	0.069	1	0.069	0.28	0.6115	No
	Residual	1.70	7	0.24			
	Lack of Fit	1.70	3	0.57			
	Pure Error	0.000	4	0.000			
	Cor Total	622.13	12				
Tensile strength	Model	4.47	5	0.89	114.73	< 0.0001	Yes
	A-TiO_2_	1.41	1	1.41	180.54	<0.0001	Yes
	B-PVA Fiber	0.66	1	0.66	84.08	<0.0001	Yes
	AB	0.039	1	0.039	5.04	0.0597	No
	A^2^	2.09	1	2.09	267.54	<0.0001	Yes
	B^2^	9.939E−003	1	9.939E−003	1.27	0.2960	No
	Residual	0.055	7	7.796E−003			
	Lack of Fit	0.055	3	0.018			
	Pure Error	0.000	4	0.000			
Weight loss	Model	16.97	5	3.39	83.55	< 0.0001	Yes
	A- TiO_2_	1.67	1	1.67	41.14	0.0004	Yes
	B-PVA Fiber	8.15	1	8.15	200.73	< 0.0001	Yes
	AB	0.22	1	0.22	5.30	0.0549	No
	A^2^	5.74	1	5.74	141.41	< 0.0001	Yes
	B^2^	0.020	1	0.020	0.49	0.5055	No
	Residual	0.28	7	0.041			
	Lack of Fit	0.28	3	0.095			
	Pure Error	0.000	4	0.000			
	Cor Total	17.25	12				
CS due to acid attack	Model	609.55	5	121.91	612.82	< 0.0001	Yes
	A- TiO_2_	240.41	1	240.41	1208.48	< 0.0001	Yes
	B-PVA Fiber	95.41	1	95.41	479.61	< 0.0001	Yes
	AB	3.31	1	3.31	16.64	0.0047	Yes
	A^2^	224.82	1	224.82	1130.12	< 0.0001	Yes
	B^2^	0.22	1	0.22	1.11	0.3262	No
	Residual	1.39	7	0.20			
	Lack of Fit	1.39	3	0.46			
	Pure Error	0.000	4	0.000			
	Cor Total	610.94	12				
pH value	Model	1.08	2	0.54	189.14	< 0.0001	Yes
	A- TiO_2_	0.79	1	0.79	278.10	< 0.0001	Yes
	B-PVA Fiber	0.42	1	0.42	147.84	< 0.0001	Yes
	Residual	0.028	10	2.845E−003			
	Lack of Fit	0.028	6	4.741E−003			
	Pure Error	0.000	4	0.000			
	Cor Total	1.10	12				
RCPT	Model	6.866E+005	5	1.373E+005	70.39	< 0.0001	Yes
	A- TiO_2_	8728.20	1	8728.20	4.47	0.0722	No
	B-PVA Fiber	1.405E+005	1	1.405E+005	72.02	< 0.0001	Yes
	AB	3669.10	1	3669.10	1.88	0.2126	No
	A^2^	5.385E+005	1	5.385E+005	276.02	< 0.0001	Yes
	B^2^	2834.12	1	2834.12	1.45	0.2673	No
	Residual	13,656.97	7	1951.00			
	Lack of Fit	13,656.97	3	4552.32			
	Pure Error	0.000	4	0.000			
	Cor Total	7.003E+005	12				
Expansion	Model	1.505E−005	5	3.010E−006	536.63	< 0.0001	Yes
	A- TiO_2_	6.146E−006	1	6.146E−006	1095.67	< 0.0001	Yes
	B-PVA Fiber	1.694E−006	1	1.694E−006	301.97	< 0.0001	Yes
	AB	9.253E−008	1	9.253E−008	16.50	0.0048	Yes
	A^2^	6.057E−006	1	6.057E−006	1079.86	< 0.0001	Yes
	B^2^	4.165E−009	1	4.165E−009	0.74	0.4174	No
	Residual	3.926E−008	7	5.609E−009			
	Lack of Fit	3.926E−008	3	1.309E−008			
	Pure Error	0.000	4	0.000			
	Cor Total	1.509E−005	12				
CS due to sulfate attack	Model	612.14	5	122.43	1052.04	< 0.0001	Yes
	A- TiO_2_	251.11	1	251.11	2157.78	< 0.0001	Yes
	B-PVA Fiber	108.45	1	108.45	931.89	< 0.0001	Yes
	AB	2.10	1	2.10	18.08	0.0038	Yes
	A^2^	200.62	1	200.62	1723.91	< 0.0001	Yes
	B^2^	0.021	1	0.021	0.18	0.6862	No
	Residual	0.81	7	0.12			
	Lack of Fit	0.81	3	0.27			
	Pure Error	0.000	4	0.000			
	Cor Total	612.96	12				
Apparent porosity	Model	57.78	5	11.56	151.90	< 0.0001	Yes
	A-TiO_2_	4.95	1	4.95	65.02	< 0.0001	Yes
	B-PVA Fiber	18.14	1	18.14	238.52	< 0.0001	Yes
	AB	0.33	1	0.33	4.32	0.0761	No
	A^2^	33.30	1	33.30	437.71	< 0.0001	Yes
	B^2^	0.021	1	0.021	0.28	0.6117	No
	Residual	0.53	7	0.076			
	Lack of Fit	0.53	3	0.18			
	Pure Error	0.000	4	0.000			
	Cor Total	58.31	12				

A prototype or model factor is considered significant if its frequency falls below 5%. Pending this, each generated model possesses statistical significance since its probability is less than 0.05. The statistical significance of the model elements A, B, AB, A^2^, and B^2^ pertains to the compressive strength model. Therefore, A, B, AB, A^2^, and B^2^ are the crucial model parameters for the TS, AP, CS due to sulphate attack, pH test, and CS due to acid attack models, respectively. Consequently, the most consequential model parameters for the weight loss and expansion models are denoted as A and B, respectively. The determination coefficient (R^2^) serves as a substantial indicator of performance. The R^2^ coefficient, represented as a percentage ranging from 0 to 1, assesses the degree of correspondence between the model and the empirical data (0–100%). The fit is more optimal when the value is greater, and conversely, when the value is lower. R^2^ and additional model evaluation variables are detailed in Table 4. R^2^ values for the following variables for each model: CS, TS, weight loss, CS resulting from sulphate attack, RCPT, weight gain, expansion, CS due to acid attack, pH test, and AP ranges from 97 to 99% respectively. Furthermore, "Adeq. Precision" performs the computation of the signal-to-noise ratio. A ratio exceeding four is preferred^97^. The Adeq precision values for the following variables are presented in Table 4. The results of this study indicate that the models demonstrate proficiency and can be effectively employed to predict reactions.

6 $$CS = + 58.52 - 5.85 \times A - 3.84 \times B - 1.01 \times AB - 8.69 \times A^{2} - 0.15 \times B^{2}$$

7 $$TS = + 5.35 - 0.44 \times A - 0.31 \times B - 0.092 \times AB - 0.85 \times A^{2} - 0.058 \times B^{2}$$

8 $$Weight Loss = + 7.43 + 0.47 \times A + 1.11 \times B - 0.22 \times AB + 1.41 \times A^{2} - 0.083 \times B^{2}$$

9 $$CS due to Acid Attack = + 54.55 - 5.69 \times A - 3.80 \times B - 0.84 \times AB - 8.82 \times A^{2} + 0.28 \times B^{2}$$

10 $$pH = + 10.12 - 0.32 \times A + 0.25 \times B$$

11 $$RCPT = + 1091.04 + 34.26 \times A + 145.73 \times B - 28.09 \times AB + 431.50 \times A^{2} - 31.10 \times B^{2}$$

12 $$Expansion = + 0.0042 + 0.0009 \times A + 0.0005 \times B + 0.00014 \times AB + 0.0015 \times A^{2} + 0.00004 \times B^{2}$$

13 $$CS due to Sulfate Attack = + 56.19 - 5.81 \times A - 4.05 \times B - 0.67 \times AB - 8.33 \times A^{2} + 0.084 \times B^{2}$$

14 $$AP = + 13.83 + 0.82 \times A + 1.66 \times B + 0.27 \times AB + 3.39 \times A^{2} - 0.086 \times B^{2}$$

**Table 4:** Models Validation Parameters.

Model Validation constraints	CS	TS	Weight loss	CS due Acid Attack	pH	RCPT	AP	Expansion	CS due to Sulphate attack
Std. Dev	0.49	0.088	0.20	0.45	0.053	44.17	0.28	7.489E−005	0.34
Mean	53.31	4.82	8.15	49.50	10.10	1326.46	15.77	5.008E−003	51.37
C.V. %	0.92	1.83	2.47	0.90	0.53	3.33	1.75	1.50	0.66
PRESS	13.99	0.40	2.38	8.85	0.056	1.202E+005	3.43	2.351E−007	6.77
-2 Log Likelihood	10.46	− 34.26	− 12.80	7.85	− 42.73	127.33	− 4.64	− 218.14	0.88
R^2^	0.9973	0.9879	0.9835	0.9977	0.9742	0.9805	0.9909	0.9974	0.9987
Adj R^2^	0.9953	0.9793	0.9717	0.9961	0.9691	0.9666	0.9843	0.9955	0.9977
Pred R^2^	0.9775	0.9106	0.8623	0.9855	0.9494	0.8283	0.9412	0.9844	0.9890
Adeq Precision	69.391	33.436	28.398	75.710	44.187	24.298	41.556	68.970	98.850

**Fig. 15:**
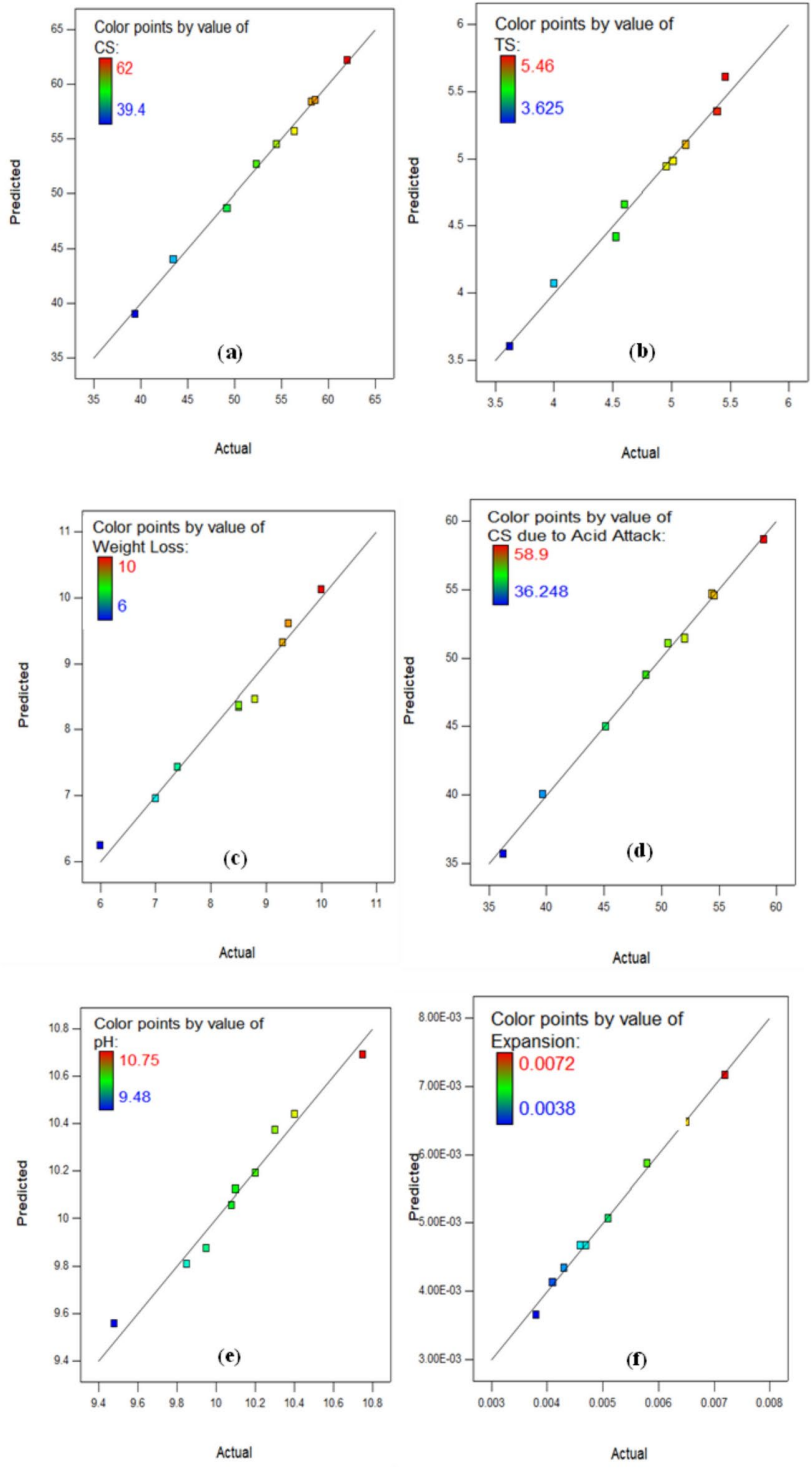

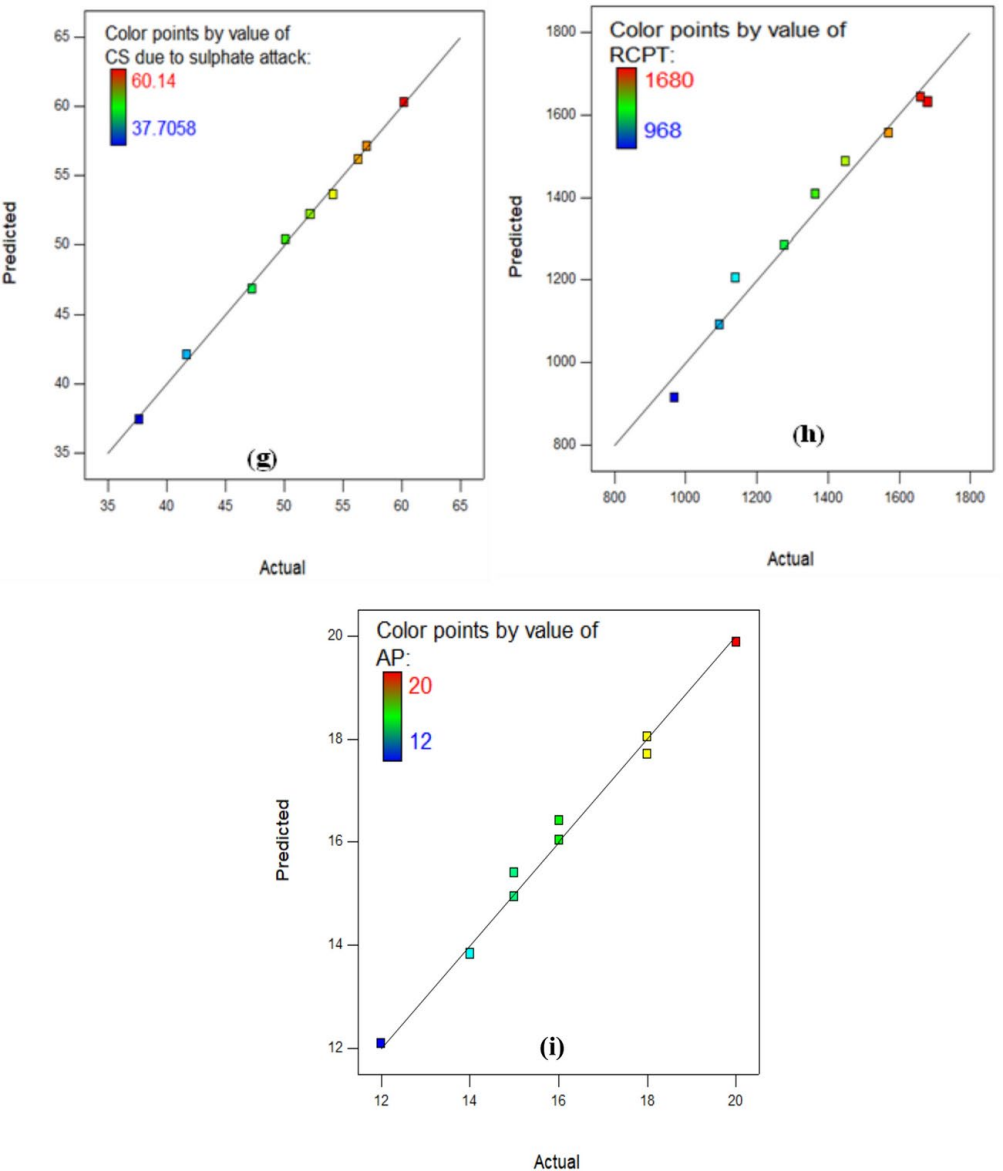
Actual against Predicted Plots for (**a**) CS, (**b**) TS, (**c**) Weight Loss, (**d**) CS due to acid attack, (**e**) pH Value, (**f**) Expansion, (**g**) CS due to Sulphate attack, (**h**) RCPT, and (**i**) AP.

The Actual versus Predicted Plot as revealed in Fig. 15a–i, which contrasts the performance of the developed models for CS, TS, weight loss, CS due acid attack, pH assessment, expansion, CS due to SA, RCPT, and AP are utilized as one of the model diagnostic tools in RSM to evaluate the quality and appropriation of the created models. In order to achieve a proper fit, the data points must be aligned with 45˚ appropriate line, which has narrow confidence level. The excellent relationship between the investigational information and the predicted outcomes from the approaches is demonstrated by the fact that the data points are precisely aligned with the line of fit in the actual versus predicted graphs for each of the developed models.

**Fig. 16:**
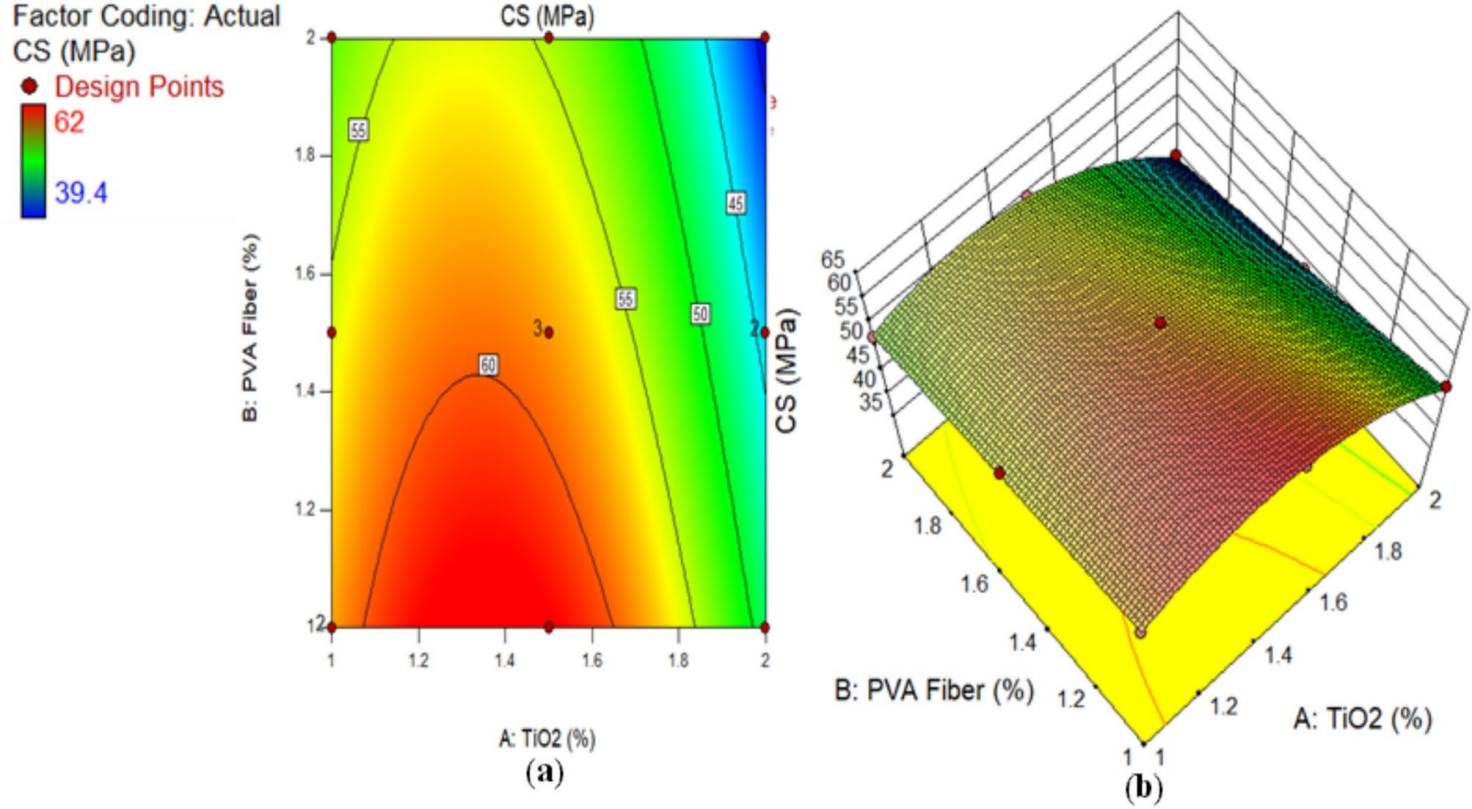
(**a**) 2D graph and (**b**) 3D graph for CS using Design Expert Software Version 10.

**Fig. 17:**
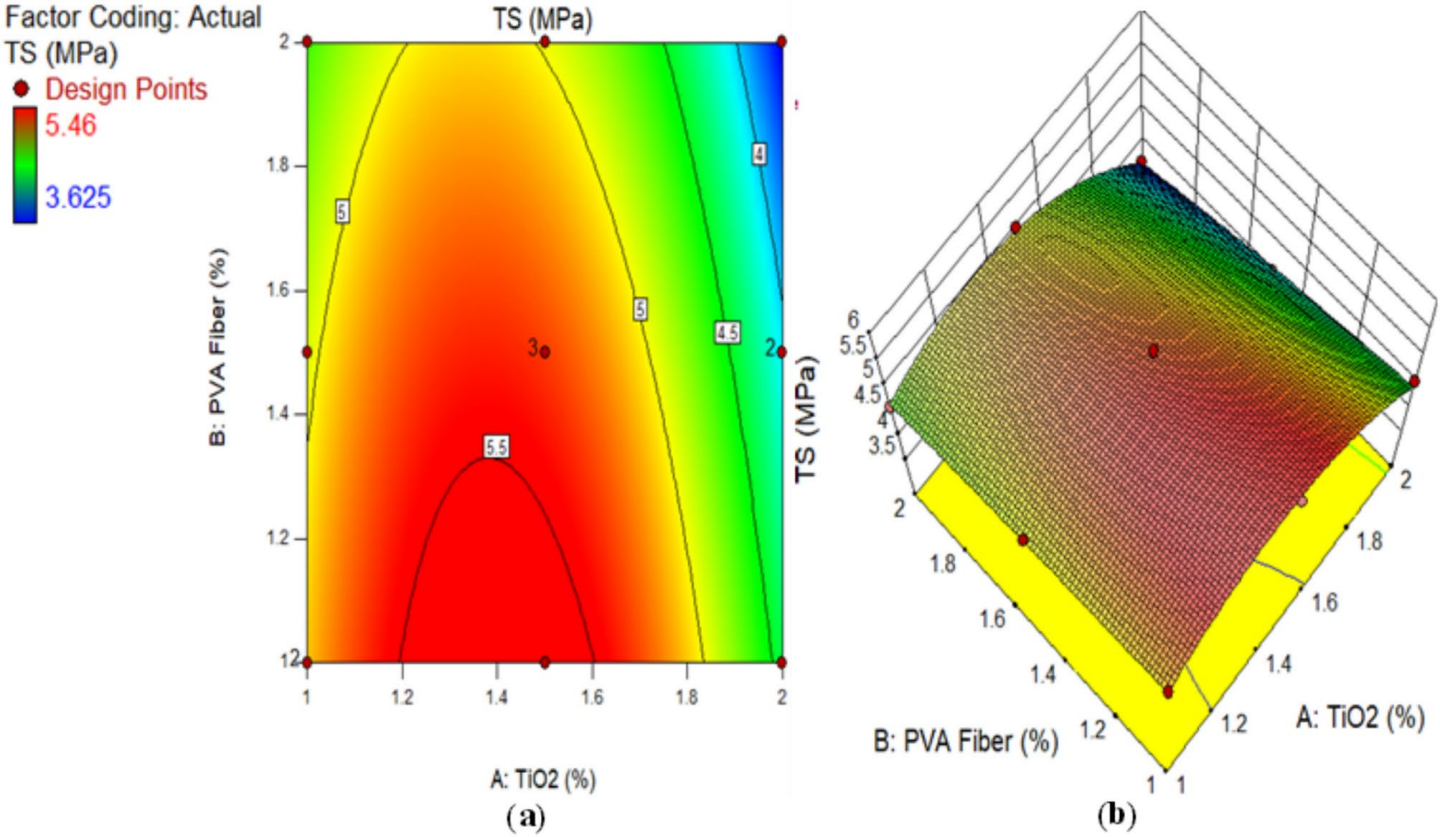
(**a**) 2D graph and (**b**) 3D graph for TS using Design Expert Software Version 10.

**Fig. 18:**
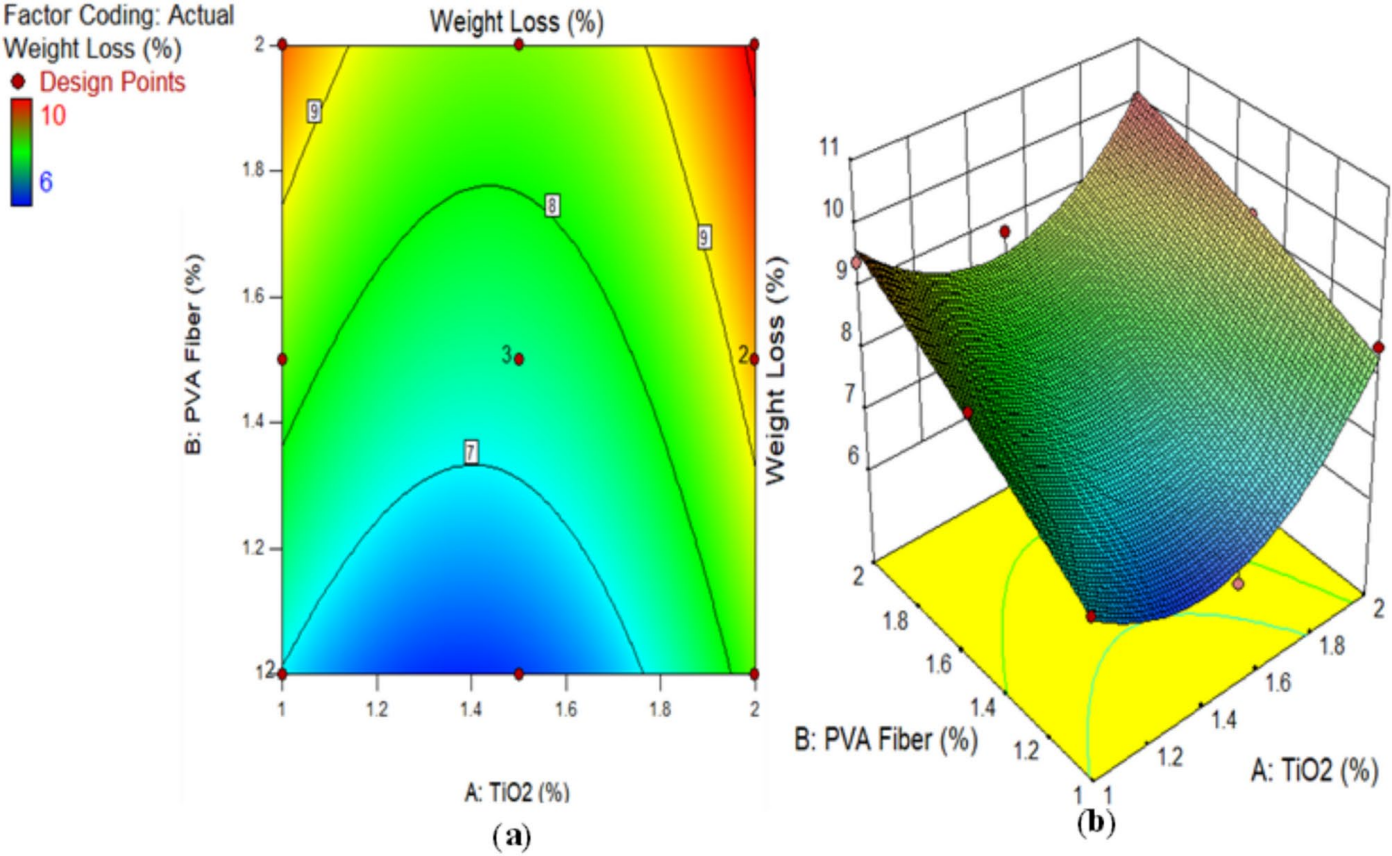
(**a**) 2D graph and (**b**) 3D graph for weight loss using Design Expert Software Version 10.

**Fig. 19:**
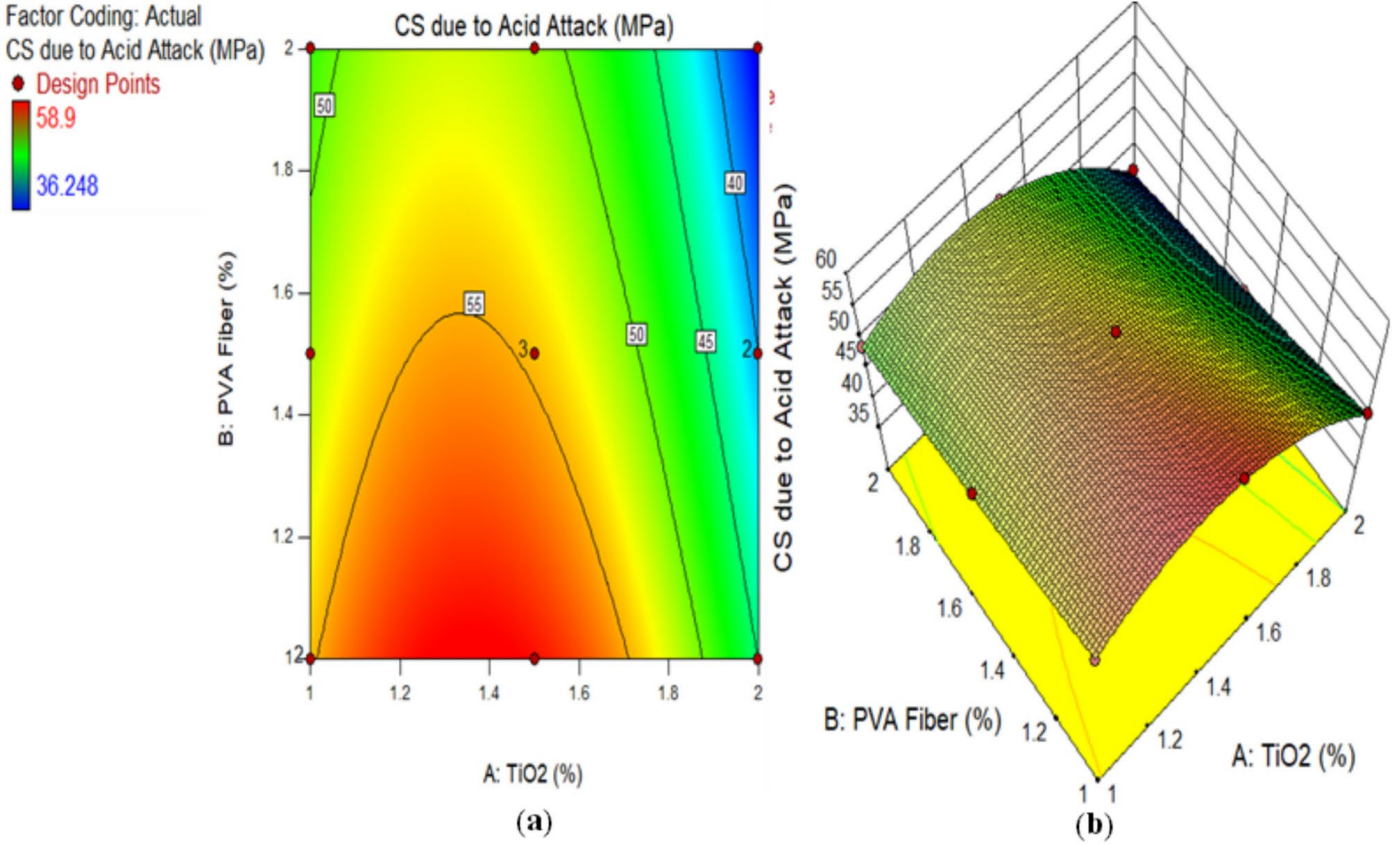
(**a**) 2D graph and (**b**) 3D graph for CS due Acid Attack using Design Expert Software Version 10.

**Fig. 20:**
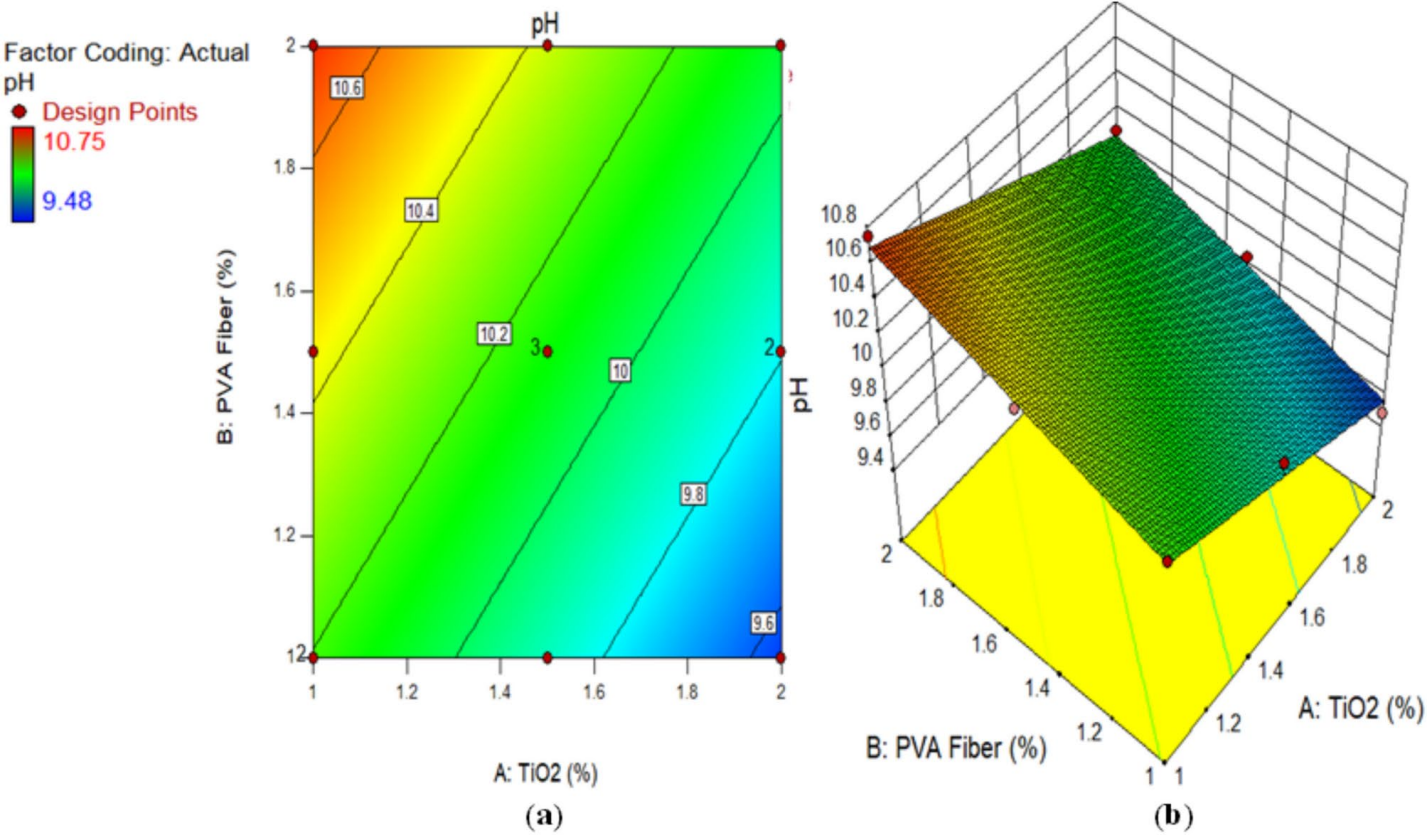
(**a**) 2D graph and (**b**) 3D graph for pH Test using Design Expert Software Version 10.

**Fig. 21:**
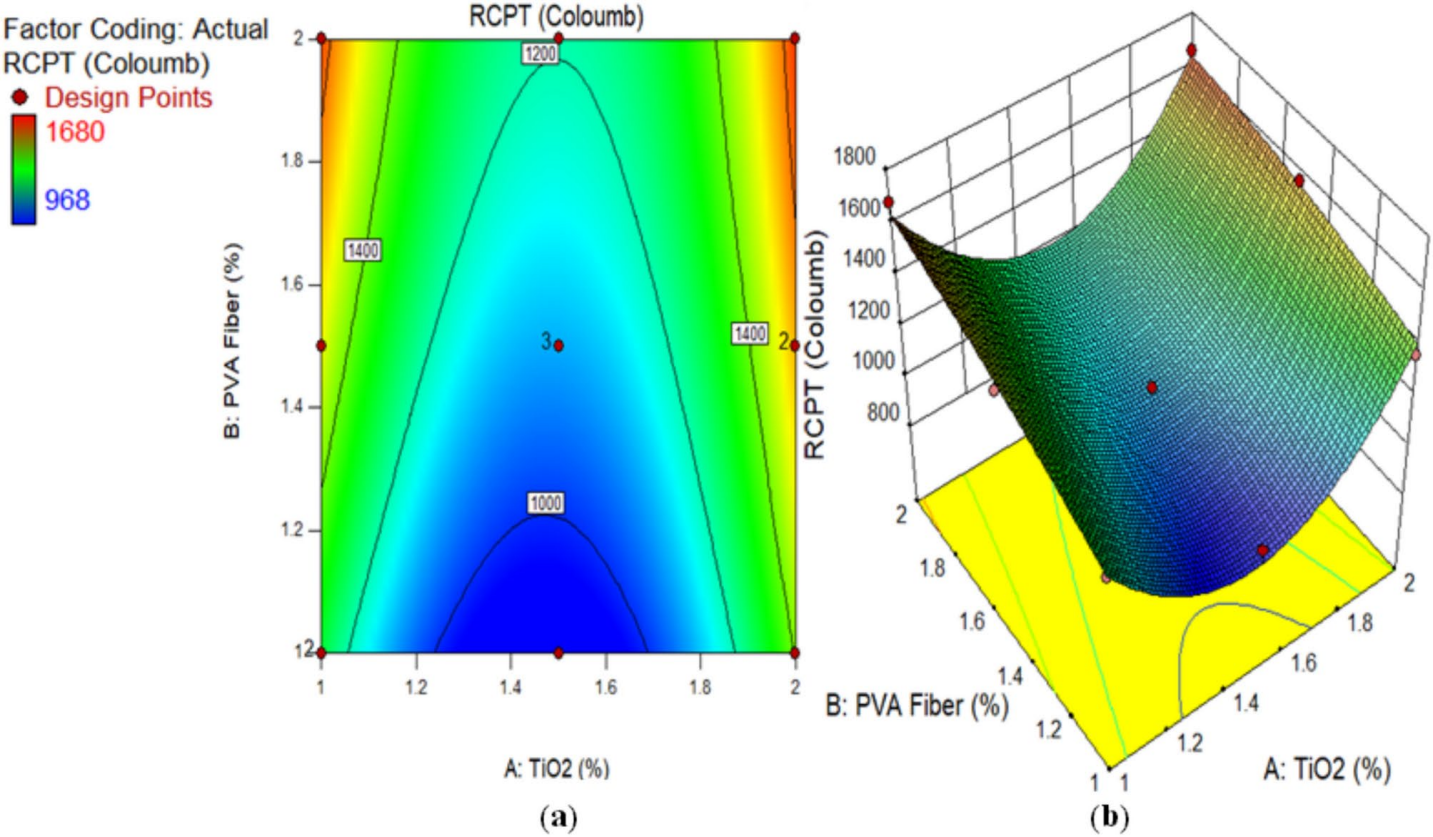
(**a**) 2D graph and (**b**) 3D graph for RCPT using Design Expert Software Version 10.

**Fig. 22:**
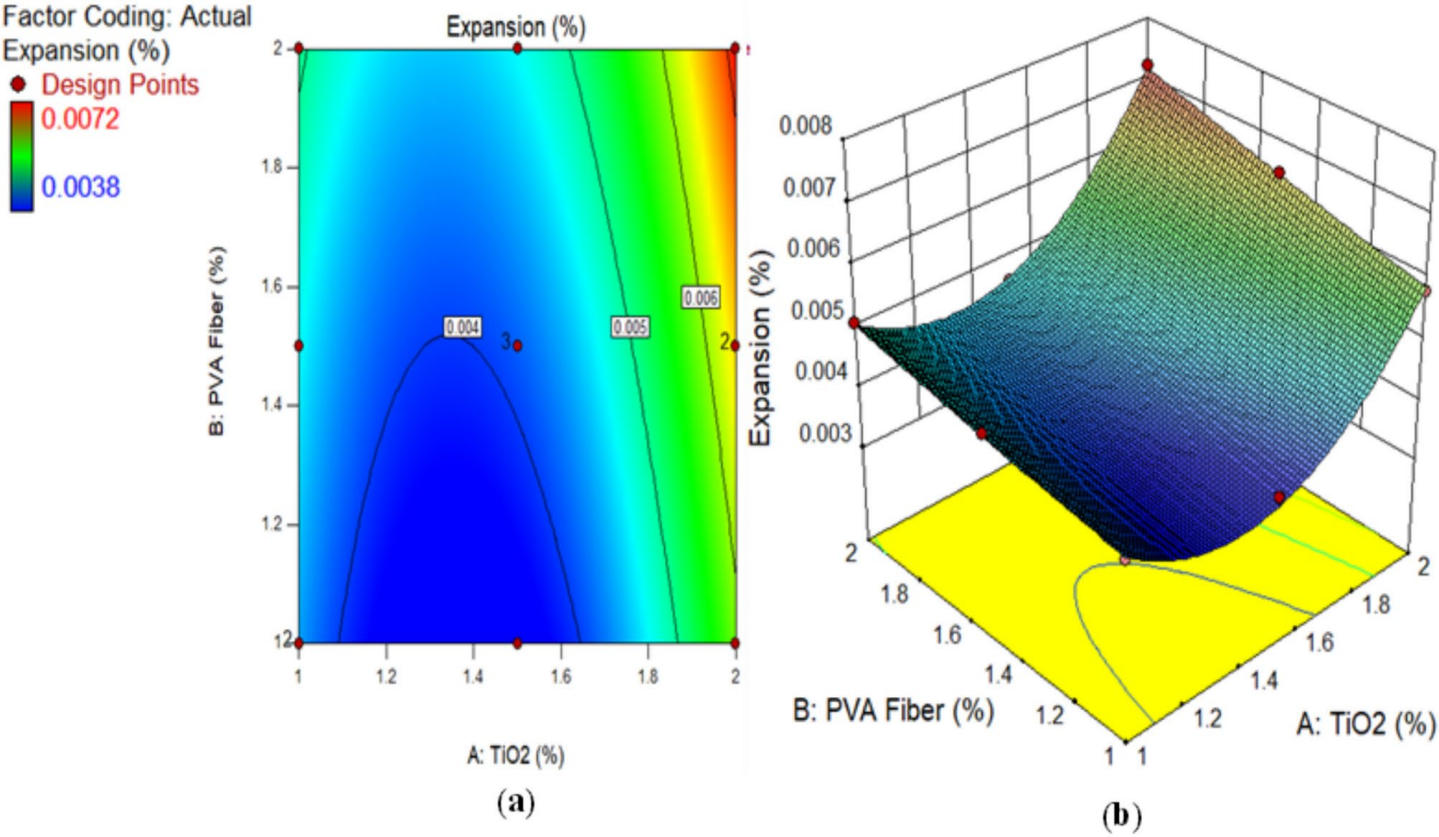
(**a**) 2D graph and (**b**) 3D graph for expansion using Design Expert Software Version 10.

**Fig. 23:**
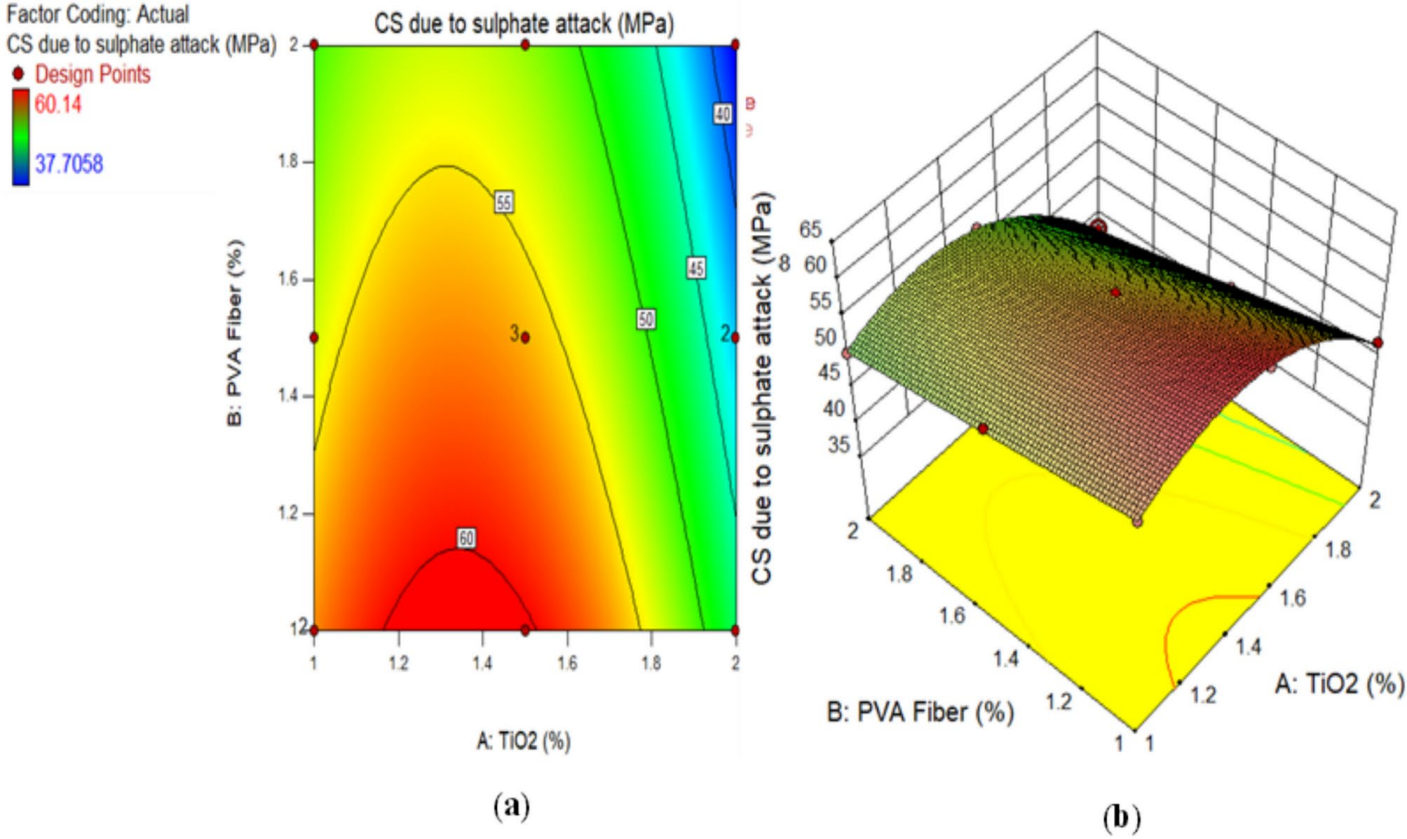
(**a**) 2D graph and (**b**) 3D graph for CS due to SA using Design Expert Software Version 10.

**Fig. 24:**
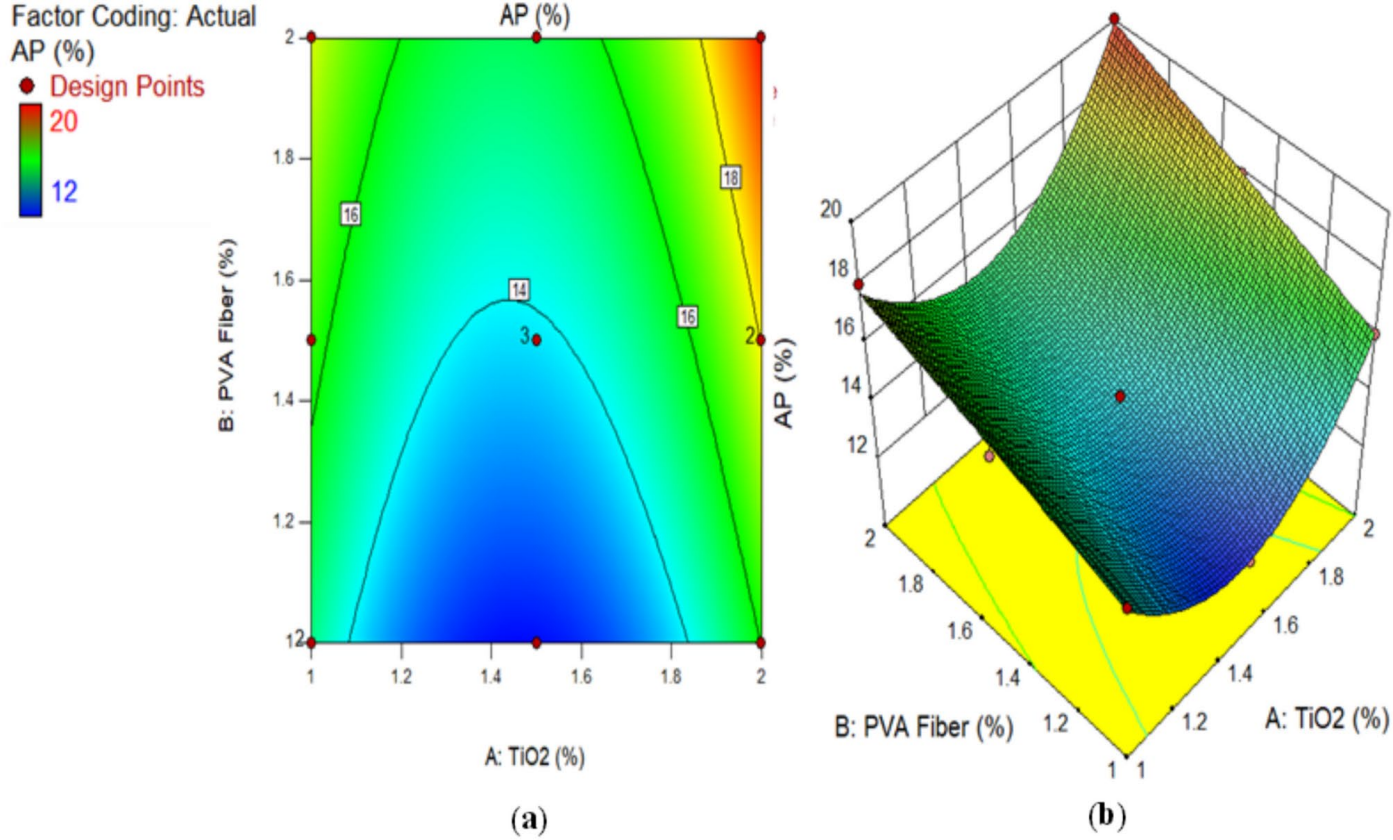
(**a**) 2D graph and (**b**) 3D graph for AP using Design Expert Software Version 10.

Alternatively, a 2D graph or a 3D graph can provide a more lucid representation of the effect of the interaction between the variables on the response. Identical to the 2D contour plot, but from a three-dimensional standpoint, is the 3D response surface plot. The investigation presented in Figs. 16, 17, 18, 19, 20, 21, 22, 23 and 24 illustrates the 2D graphs and 3D diagrams for each of the developed response predictive models. In the context of 2D and three-dimensional response surface diagrams, regions which symbolise distinct intensities of the response resulting from the interaction of the input variables are denoted by colour coding. The zones denoted in red represent areas with the greatest magnitudes of responses, whereas the regions in blue correspond to areas with the least intense responses. Diagrammatically, the interactions between the input variables and their effects on the responses are in perfect accordance with the previously discussed variables’ effects on the CS, TS, RCPT, sulphate attack, acid attack tests, and porosity.

### Optimization

In order to establish the highest findings for desirable parameters, it is designed to determine the optimum values of input factors. The achievement of the target function (CS, TS, CS due to acid attack, weight loss, pH assessment, CS due to SA, expansion, RCPT, and porosity) is facilitated through the establishment of purposes for input parameters that vary in significance and condition. Using the desirability value (where 0 ≤ dj ≤ 1), the optimisation is assessed. A higher outcome is indicated by a value approaching one^98^.

**Fig. 25:**
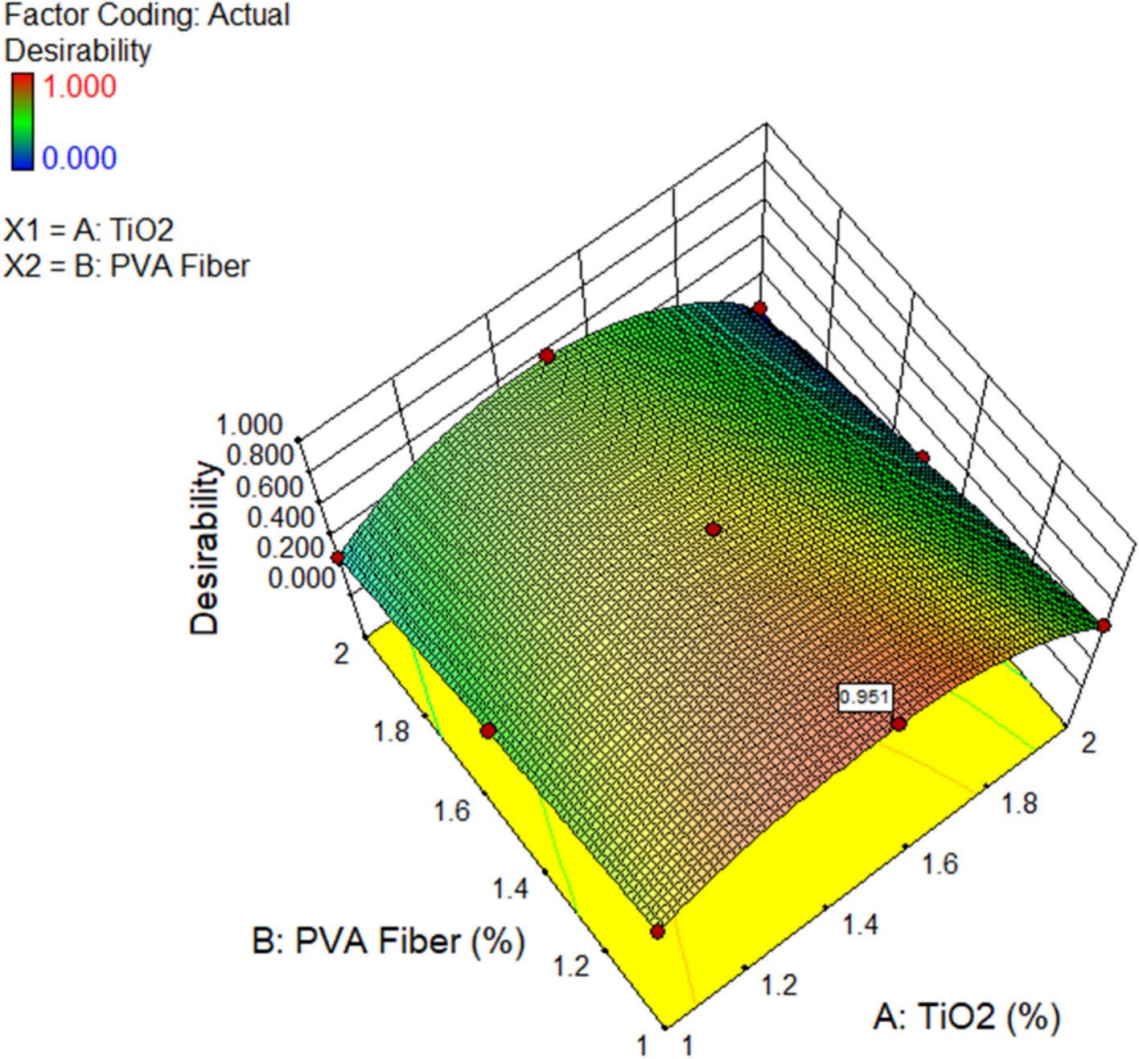
3D diagram for the desirability of the optimization using Design Expert Software Version 10.

Table 5 outlines the rationale and standards that underpin the optimisation process for this particular instance. As a result, the mechanism may ascertain the optimal quantity to achieve the specified objectives, even though the utilisation of TiO_2_ and PVA fiber has been limited to a range of 1% to 2% and 1%, 1.5%, and 2%, respectively. According to the optimisation results, the highest values for CS, TS, CS due to acid attack, weight loss, pH assessment, CS due to SA, expansion, RCPT, and porosity were attained by containing 1.489% TiO_2_ and 1% PVA fiber in composites at 62.30 MPa, 5.62 MPa, 58.7 MPa, 6.22%, 9.88, 60.42 MPa, 0.0036%, 913.01 Coulombs, and 12.08% consistently. The degree to which the proposed solution and the actual result are comparable establishes the criteria for desirability. For optimum results, it is recommended to approach one with a desirability. Optimising the response is feasible, as indicated by the desirability value of 0.951. The 3D diagram for desirability is illustrated in the Fig. 25.

**Table 5:** Optimization TiO_2_ results.

Factors	Input factors						Responses (output factors)				
	TiO_2_ (%)	PVA (%)	CS (MPa)	TS (MPa)	AP (%)	RCPT (Coulombs)	Sulphate attack		Acid attack		
							Expansion (%)	CS (MPa)	Wt. loss (%)	CS	pH
Value											
Min.	1	1	39.40	3.625	12	968	0.0038	37.71	6	36.24	9.48
Max.	2	2	62	5.46	20	1680	0.0072	60.14	10	58.90	10.75
Goal	Range	Range	Max	Max	Min	Min	Min	Max	Min	Max	Min
Optimization Results	1.489	1	62.30	5.62	12.08	913.01	0.0036	60.43	6.22	58.72	9.88
Desirability	95.10% (0.951)										

## Conclusion

The objective of this study was to investigate the impact of TiO_2_ nanoparticles on the durability characteristics of ECC accumulated with 1–2% PVA fiber. Following the investigation, the key points were reached:

- At 1.50% TiO_2_ nanoparticles in ECC, the optimal strength of composites accumulation with 1% PVA fiber is observed, whereas the minimal strength of composites accumulation with 1% PVA fiber is measured at 2% TiO_2_ nanoparticles. It has been suggested that the strongest ECCs are produced with a 1.50% TiO_2_ by weight of PC addition, and that as more TiO_2_ accumulates in ECC, the strength starts to decline.
- The resistance to sulphate assault is attributed to the pozzolanic characteristics of TiO_2_ particles, which fill spaces in pores and hinder the entrance of ions. Increased quantities of TiO_2_ improve the stability of ECC in harsh environments, with increased TiO_2_ content leading to better resistance to weight loss and dimensional changes.
- The production of ECC comprising varying concentrations of TiO_2_ as nanoparticles is aided by the increased use of PVA fibers; however, the observed all-pH values are lesser than CM. Furthermore, it has been detected that the pH of an ECC mixture containing PVA fiber in different proportions reduces as the amount of TiO_2_ rises.
- More resistance to chloride ion penetration was attained through the utilisation of TiO_2_ nanoparticles. The chloride permeability values exhibited a decrease upon the introduction of nanoparticles of TiO_2_. All mixtures were categorised as having extremely low chloride permeability, apart from the control mixture which were categorised as having low permeability.
- The porosity of ECC exhibited a decrease upon the introduction of nanoparticles of TiO_2_ while the porosity of ECC increases as increasing the quantity of fiber. Moreover, the porosity of all mixtures containing TiO_2_ and fiber were lower than control mixture.
- To prediction the CS, RCPT, acid resistance, and sulphate resistance of ECC at the 28-day mark, RSM models were developed, with the accuracy contingent upon the ratios of PVA fibers and TiO_2_ utilised as nanomaterials. The difference between adjusted R^2^ to predicted R^2^ is less than 0.2 and confidence level of 95% for each model.
- By incorporating 1.50% TiO_2_ in composites combined with 1% PVA fiber, the challenges associated with large-scale ECC implementation, and the detrimental effects of sulphate and acid attack may be mitigated. As a result, its implementation in the practical sectors of the construction industry is advised.

## Future research

The integration of TiO₂ and polyvinyl alcohol (PVA) fibers in concrete offers a viable method for improving durability against harsh circumstances, including sulphate and acid attacks. Future research endeavours may concentrate on the following domains:

- Perform comprehensive experimental investigations to assess the long-term efficacy of TiO₂ and PVA fiber-reinforced concrete subjected to cyclic wet-dry and freeze–thaw circumstances in sulphate and acidic environments.
- Evaluate the time stability of TiO₂’s photocatalytic performance and its impact on alleviating degradation.
- Investigate the synergistic effects of TiO₂ and PVA fibers on fracture resistance and self-healing properties in concrete subjected to sulphate and acid attacks.
- Assess the ecological consequences of incorporating TiO₂ and PVA fibers using life cycle assessments (LCA) and explore alternate sources or recycling methods for these materials.
- Examine the feasibility of integrating TiO₂ and PVA fibers with supplemental cementitious materials (SCMs) to enhance durability and minimize the carbon impact.

## Data Availability

The datasets used and/or analyzed during the current study are available from the corresponding author upon reasonable request.
